# Incidence and risk factors for myocardial injury after laparoscopic adrenalectomy for pheochromocytoma: A retrospective cohort study

**DOI:** 10.3389/fonc.2022.979994

**Published:** 2022-09-12

**Authors:** Ling Lan, Qian Shu, Chunhua Yu, Lijian Pei, Yuelun Zhang, Li Xu, Yuguang Huang

**Affiliations:** ^1^ Department of Anesthesiology, Peking Union Medical College Hospital, Chinese Academy of Medical Science and Peking Union Medical College, Beijing, China; ^2^ State Key Laboratory of Complex Severe and Rare Diseases, Peking Union Medical College Hospital, Chinese Academy of Medical Science and Peking Union Medical College, Beijing, China; ^3^ Medical Research Center, Peking Union Medical College Hospital, Chinese Academy of Medical Science and Peking Union Medical College, Beijing, China

**Keywords:** catecholamine, hypertension, injury, pheochromocytoma, tachycardia

## Abstract

**Background:**

Pheochromocytoma is a rare catecholamine-secreting tumor. Tumor resection remains a high-risk procedure due to intraoperative hemodynamic instability nowadays, which may lead to myocardial injury. We aimed to determine the incidence and associated risk factors for myocardial injury after laparoscopic adrenalectomy for pheochromocytoma.

**Methods:**

Adult patients (n=350, American Society of Anesthesiology physical status 1–3) who underwent elective laparoscopic adrenalectomy for pheochromocytoma under general anesthesia between January 31, 2013 and January 31, 2020 were included in this observational, retrospective, single-center, cohort study. Blood troponin I levels were measured before and during the first 2 days after surgery. Myocardial injury was defined as an elevated troponin I level exceeding the 99^th^ percentile upper reference limit due to cardiac ischemic causes.

**Results:**

Myocardial injury occurred in 42/350 patients (12.0%, 95% confidence interval: 9.0%–15.9%). In multivariable analysis (adjusted odds ratios [95% confidence intervals]), previous ischemic heart disease or stroke (5.04 [1.40–18.08]; *P*=0.013), intraoperative heart rate >115 bpm (2.55 [1.06–6.12]; *P*=0.036), intraoperative systolic blood pressure >210 mmHg (2.38 [1.00–5.66]; *P*=0.049), and perioperative decrease in hemoglobin level(1.74 [1.15–2.64] per g/dL decrease; *P*=0.008) were associated with an increased risk of myocardial injury. For the cumulative duration of dichotomized intraoperative hemodynamics, multivariable analysis showed that intraoperative heart rate >115 bpm for >1 minute (2.67 [1.08–6.60]; *P*=0.034) and systolic blood pressure >210 mmHg for >1 minute (3.78 [1.47–9.73]; *P*=0.006) were associated with an increased risk of myocardial injury. The risk of myocardial injury progressively increased with a longer cumulative duration of intraoperative tachycardia and hypertension.

**Conclusions:**

There is a high incidence of myocardial injury after laparoscopic adrenalectomy for pheochromocytoma. The identified risk factors may assist physicians in detecting high-risk patients and providing guidance for intraoperative hemodynamics and perioperative hemoglobin management.

## Introduction

Pheochromocytomas are rare catecholamine-secreting tumors arising from adrenomedullary chromaffin cells, with an overall incidence of 0.18 to 0.66 per 100,000 person-years in the general population (1–3) and 0.2% to 0.6% of hypertensive patients (4). Catecholamine surge and diminution cause dramatic hemodynamic instability during tumor resection, leading to catastrophic cardiovascular complications, such as myocardial infarction, life-threatening ventricular arrhythmias, and Takotsubo-like cardiomyopathy (5–7). Recently severe cardiovascular complications decreased significantly because of early diagnosis and surgically resection following optimal medical preparation (8–11). Furthermore, the utilization of laparoscopic approaches (12, 13) has also improved postoperative adverse outcomes over the last two decades. However, the incidence of intraoperative hemodynamic instability remains relatively high during laparoscopic adrenalectomy for pheochromocytoma (14, 15), which may cause myocardial injury after surgery.

Postoperative myocardial injury, defined as troponin elevation due to ischemic etiologies, has recently been identified as a crucial and undetected adverse cardiovascular outcome following non-cardiac surgeries (16). Troponin elevation has also been strongly associated with higher morbidity and 30-day mortality (17–20). Previous studies (21–24) have reported the perioperative risk factors of myocardial injury after non-cardiac surgeries. In addition to intraoperative heart rate (HR) and blood pressure (BP), patient characteristics are also associated (16, 22). Currently, limited data exist on myocardial injury after laparoscopic adrenalectomy for pheochromocytoma. Therefore, we conducted this observational, retrospective, single-center, cohort study to determine the incidence and risk factors for myocardial injury after laparoscopic adrenalectomy for pheochromocytoma and we evaluated the associations and thresholds of intraoperative BP and HR with myocardial injury in this population especially.

## Materials and methods

### Study design and participants

**Figure 1:**
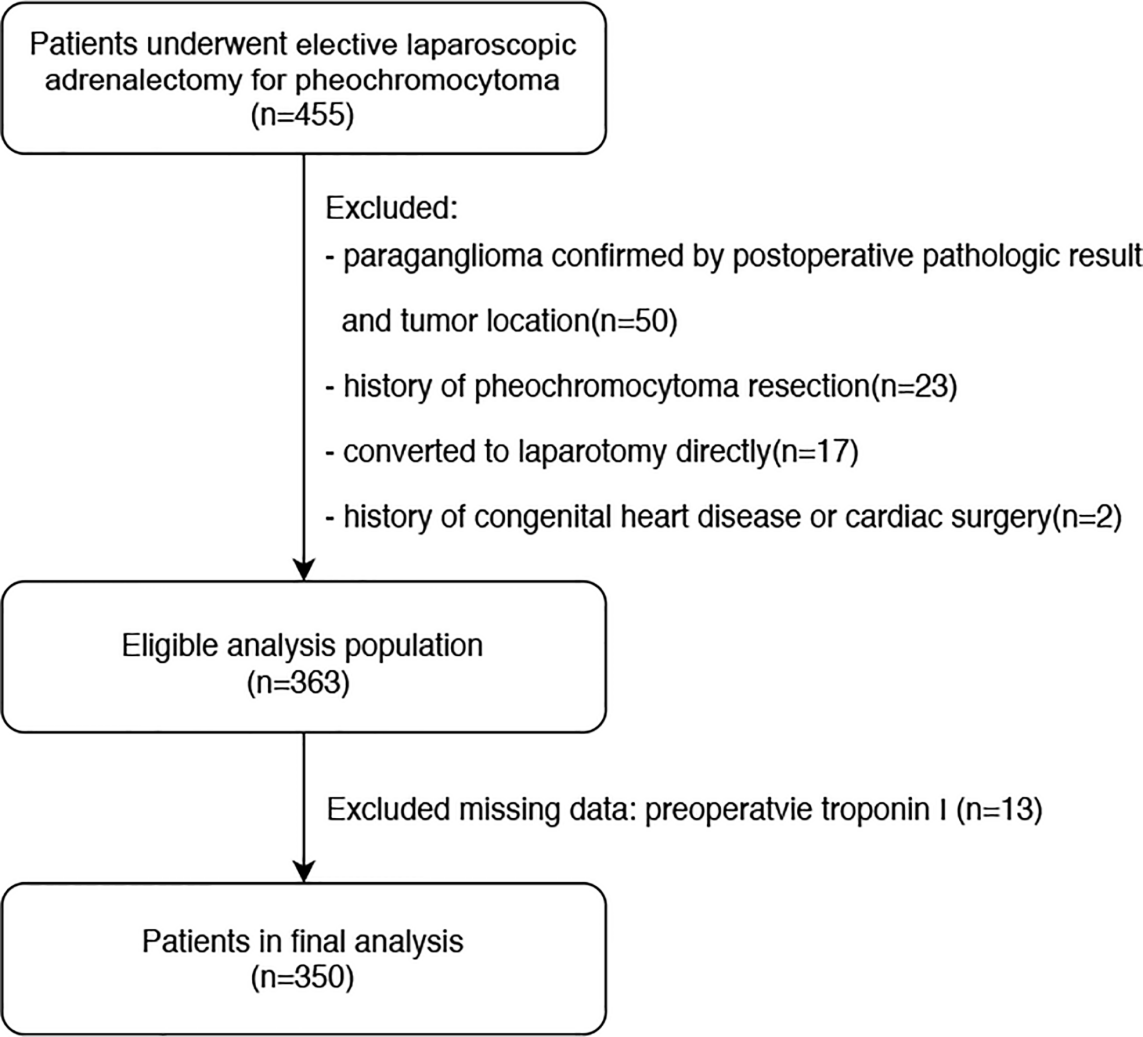
Flow diagram of patient selection.

The manuscript was prepared according to the Strengthening the Reporting of Observational Studies in Epidemiology statement (25). This was an observational, retrospective, single-center, cohort study conducted at Peking Union Medical College Hospital (PUMCH), Beijing, China. The study design was approved by the Research Ethics Commission of PUMCH (approval number: S-K1878). The requirement for written informed consent was waived owing to the retrospective nature of the study. We retrospectively reviewed the electronic medical records of 455 patients who underwent elective laparoscopic adrenalectomy for pheochromocytoma under general anesthesia at our institution between January 31,2013 and January 31, 2020. Pheochromocytoma was diagnosed based on the postoperative pathological result combined with tumor location, as evaluated using preoperative abdominal imaging. Initially, 92 patients were excluded due to being confirmed with paraganglioma on tumor location(50 cases), history of pheochromocytoma or paraganglioma resection(23 cases),undergoing laparotomy directly rather than laparoscopic surgery(17 cases), and history of congenital heart disease or cardiac surgery (2 cases). From there, 363 patients were eligible for preliminary analysis, but 13 more cases were excluded due to missing troponin I values before or within 2 days after surgery. Ultimately, 350 patients were included in the final statistical analysis. A flowchart of the patient selection process is shown in **Figure 1**.

For preoperative management, all patients received non-selected α-blockade therapy for more than seven days. Phenoxybenzamine was administered and the dose was titrated to maintain the BP < 140/90 mm Hg. The β-blocker was added when necessary to maintain the HR < 90 bpm after α-blockade had been achieved. Treatment also included a high-sodium diet and fluid intake to reverse catecholamine-induced blood volume contraction before surgery to prevent severe hypotension after tumor removal.

For anesthesia implement and critical care, all patients received general anesthesia and an arterial line for invasive BP monitoring before general anesthesia implement. Central venous access was retained after anesthesia induction. Arterial BP and HR measurements were recorded automatically by the monitor every 10 seconds. During surgery, hypertensive and hypotensive episodes were treated at the anesthesiologists’ discretion. Generally, vasodilators were administered when systolic blood pressure (SBP) was higher than 160 mmHg and vasopressors were administered when SBP was lower than 90 mmHg. Hypertension was treated with vasodilators, such as nitroprusside infusion and phentolamine bolus for target BP response. Hypotension was treated with aggressive administration of intravenous fluid, discontinuation of vasodilator infusions, and administration of rapidly acting vasopressors, including phenylephrine, norepinephrine, and epinephrine. Esmolol was administered for tachycardia control. After surgery, all patients were routinely transferred to the intensive care unit before admission to the general ward. Blood troponin I levels were measured before and during the first 2 days after surgery. For patients with increased troponin I level, a cardiology specialist was consulted initially. At the same time, a 12-lead electrocardiogram(ECG) and bedside point-of-care echocardiography were performed to identify evidence of myocardial infarction. The troponin I test was repeated every 2–6 hours following the cardiology specialists’ consultation until it returned to normal levels. Myocardial infarction diagnoses were centrally adjudicated and required both, the increased troponin I levels and at least one symptom (chest pain or shortness of breath) or sign (ECG or echocardiogram abnormality). Once the patient was diagnosed with myocardial infarction, cardiovascular treatment was initiated immediately, including antiplatelet therapy, anticoagulation, catheterization, and angioplasty.

### Study procedures

Preoperative, intraoperative and postoperative data were extracted from electronic medical records. Preoperative data included age, sex, body mass index(BMI), American Society of Anesthesiology(ASA) physical status, smoking history, alcohol use, diabetes mellitus, hypertension, previous ischemic heart disease or stroke, previous congestive heart failure, preoperative hemoglobin, troponin I and creatine levels, 24-hour urinary catecholamine level, tumor size and location, preoperative medical treatment, as well as noninvasive BP and HR before surgery. Ischemic heart disease refers to a history of myocardial infarction, positive exercise test, current complaint of ischemic chest pain or nitrate use, or ECG with pathological Q waves; it also includes patients with a history of previous coronary bypass surgery or angioplasty. Stroke refers to the ischemic stroke (thrombotic/embolic/systemic hypoperfusion) or hemorrhagic stroke (intracerebral hemorrhage, subarachnoid hemorrhage). Intraoperative data included hemodynamic parameters (HR, SBP, diastolic blood pressure [DBP]), surgical approach and duration, estimated blood loss, red blood cell (RBC) and fresh frozen plasma (FFP) transfusion. Postoperative data included postoperative hemoglobin and troponin I levels of the first 2 days after surgery, postoperative hypotension requiring vasopressor infusion, length of stay, in-hospital complications and mortality.

Artifacts of intraoperative hemodynamic parameters (HR, SBP, DBP) were removed using published rules (22), in the following order (1): BP and HR measurements documented as artifacts (2); BP and HR measurements beyond the defined range: (a) SBP ≥300 mmHg or SBP ≤20 mmHg, (b) SBP ≤ DBP + 5 mmHg, or (c) HR ≥140 bpm or HR ≤40 bpm. Intraoperative tachycardia and hypertension were sorted and dichotomized according to predefined thresholds for the highest intraoperative HR (>100, >105, >110, >115, and >120 bpm) and the highest intraoperative SBP (>160, >180, >200, >210, and >220 mmHg) separately and considered as categorical variables. A perioperative decrease in hemoglobin level was defined as the difference between the preoperative and minimum hemoglobin levels within 2 days after surgery.

### Study outcomes

The primary outcome was myocardial injury occurring within 2 days after surgery. Myocardial injury was defined as an elevated troponin I level exceeding the 99^th^ percentile upper reference limit due to cardiac ischemic causes, according to the VISION study (19, 26). Myocardial injury was independently assessed by two physicians. For patients with elevated troponin I levels, study personnel searched the electronic medical records for evidence of ischemic symptoms, electrocardiogram changes, and a diagnosis of myocardial infarction. The fourth universal definition of myocardial infarction was used as the diagnostic tool for myocardial infarction (27). The secondary outcomes were other postoperative complications classified by Clavien-Dindo grade (28) and in-hospital mortality.

### Statistical analyses

Continuous numerical data are presented as mean ± standard deviation or median (interquartile range), and categorical data as frequencies and percentages. Univariable analysis was performed using the Student’s *t*-test or Wilcoxon–Mann–Whitney test for continuous variables. Categorical variables were examined using the Pearson chi-square test or Fisher’s exact test. Multivariable logistic regression analysis was used to explore the risk factors associated with myocardial injury. First, we developed a base model (Model 1) to explore myocardial injury without intraoperative hemodynamics. Variables potentially related to the risk of myocardial injury were also included. These included age, sex, BMI, ASA physical status, smoking history, alcohol use, diabetes mellitus, hypertension, previous ischemic heart disease or stroke, previous congestive heart failure, preoperative creatine, preoperative 24-hour urinary catecholamine elevation (none, epinephrine, norepinephrine, dopamine), multiples of the normal reference upper limit value (24-hour urinary epinephrine, 24-hour urinary norepinephrine, 24-hour urinary dopamine), tumor location, tumor maximum diameter, preoperative medications (alpha blockade only, alpha blockade + calcium channel blocker, alpha blockade + beta blockade, alpha blockade + beta blockade + calcium channel blocker), duration of alpha blockade, daily dose of phenoxybenzamine, hemodynamic variables before surgery (HR, SBP, DBP), surgical approach and duration, RBC and FFP transfusion, perioperative decrease in hemoglobin, and postoperative hypotension requiring vasopressor infusion. Next, we estimated the potential risk factors for Model 1 following the addition of each of the five previously established HR thresholds (Model 2) and SBP thresholds (Model 3). Finally, to explore the cumulative duration of the dichotomized intraoperative hemodynamics above, we assessed the relationships between myocardial injury and the aforementioned variables for a cumulative time of 1, 5, 10, 15, and 20 minutes (Model 4).

Considering the total number of myocardial injuries (n=42) in our study and to avoid overfitting the model, four variables were chosen for multivariable analysis based on previous findings and clinical constraints. The collinearity between factors was assessed using the variance inflation factor. A variance inflation factor >5 was used to define significant collinearity (29). All statistical analyses were conducted using STATA (version 15.0; Stata Corp., TX, USA) and R 3.6.1 software (R Foundation for Statistical Computing, Vienna, Austria). A two-sided *P*-value <0.05 was considered statistically significant.

## Results

### Patients’ characteristics

A total of 350 patients who underwent elective laparoscopic adrenalectomy for pheochromocytoma were included finally. Patients’ characteristics, medical history, and perioperative data are shown in **Table 1**. Patients in our study were relatively young (mean age, 44.5 ± 13.4 years), and over half were female (54.0%). BMI was normal (23.8 kg/m^2^), and most patients were non-smokers (82.0%), with no alcohol use (81.9%) and ASA physical status 2–3 (97.4%). Although less than half of the patients had hypertension before surgery (44.9%), all patient received preoperative medical preparation, including alpha-adrenergic blockade alone (66.0%) or combination with beta blockade (24.9%), and achieved the target hemodynamics. Other comorbidities included diabetes mellitus (18.3%), previous ischemic heart disease or stroke (7.7%), and previous congestive heart failure (6.0%). The mean maximum tumor diameter was 4.8 cm, and the 24-hour urinary norepinephrine level was particularly elevated in 51.1% patients. More than 90% patients with unilateral tumors underwent successful laparoscopic resection, and 13 patients (3.7%) were converted to laparotomy during surgery. Intraoperative established blood loss was minimal (median [interquartile range]) (100 [50–300] mL). However, the postoperative decrease in hemoglobin level was significant (mean ± standard deviation) (1.8 ± 1.1 g/dL). Prolonged hypotension requiring vasopressors infusion occurred in over half of the patients (58.9%) before they were transferred to the intensive care unit.

**Table 1:** Patient characteristics based on postoperative myocardial injury.

Factors	Total(n = 350)	Myocardial injury (n = 42)	No myocardial injury (n = 308)	Mean Difference/Median Difference/OR (95% CI)	P-value
Age, years (mean ± SD)	44.5 ± 13.4	44.4 ± 14.5	44.6 ± 13.3	- 0.2	0.923
Male, n (%)	161 (46.0)	22 (52.4)	139 (45.1)	1.34 (0.70, 2.55)	0.376
BMI, kg/m2	23.8 ± 3.3	23.3 ± 3.1	23.9 ± 3.3	- 0.6	0.239
ASA physical status, n (%)					
1	9 (2.6)	0 (0.0)	9 (2.9)		*
2	142 (40.6)	15 (35.7)	127 (41.2)	0.00 (0.00, 3.80)	0.600
3	199 (56.9)	27 (64.3)	172 (55.8)	0.00 (0.00, 2.81)	0.609
Smoking history, n (%)	63 (18.0)	12 (28.6)	51 (16.6)	2.02 (0.97,4.20)	0.057
Alcohol use, n (%)	60 (17.1)	10 (23.8)	50 (16.2)	1.61(0.75, 3.49)	0.222
Diabetes, n (%)	64 (18.3)	9 (21.4)	55 (17.9)	1.25 (0.50, 2.87)	0.574
Hypertension, n (%)	157 (44.9)	20 (47.6)	137 (44.5)	1.13 (0.56, 2.28)	0.701
Previous ischemic heart disease or stroke, n (%)	27 (7.7)	8 (19.0)	19 (6.2)	3.57 (1.25, 9.34)	0.003
Previous congestive heart failure, n (%)	21 (6.0)	4 (9.5)	17 (5.5)	1.80 (0.42, 5.91)	0.298
Preoperative creatine (µmol/L)	68.2 ± 14.9	70.4 ± 17.4	67.9 ± 14.5	2.5	0.295
Preoperative 24-h urinary catecholamine elevated					
None	85(24.3)	74 (24.0)	11 (26.2)		*
Epinephrine elevated	81(23.1)	71 (23.1)	10 (23.8)	0.95(0.34,2.63)	0.908
Norepinephrine elevated	179(51.1)	159 (51.6)	20 (47.6)	0.85(0.36,2.06)	0.677
Dopamine elevated	5(1.4)	4 (1.3)	1 (2.4)	1.68(0.03,19.09)	0.520
Multiple of the normal reference upper limit value					
24-h urinary epinephrine	5.0 ± 15.0	6.9 ± 18.7	4.8 ± 14.4	2.1	0.415
24-h urinary norepinephrine	6.3 ± 10.2	7.7 ± 13.6	6.1 ± 9.6	6.6	0.360
24-h urinary dopamine	1.0 ± 1.3	0.9 ± 0.6	1.0 ± 1.3	- 0.1	0.632
Tumor location, n (%)				0.87 (0.16, 3.06)	1.000
Unilateral	322 (92)	39 (92.9)	283 (91.9)		
Bilateral	28(8)	3 (7.1)	25 (8.1)		
Maximum tumor diameter (cm)	4.8 ± 1.9	5.2 ± 2.3	4.7 ± 1.8	0.5	0.088
Preoperative medications, n (%)					
α blockade only	231(66)	24 (57.1)	207 (67.2)		*
α blockade + CCB	32(9.1)	8 (19.0)	24 (7.8)	0.35 (0.13,1.00)	0.018
α blockade + β blockade	63(18)	5 (11.9)	58 (18.8)	1.34 (0.47,4.71)	0.563
α blockade + β blockade + CCB	24(6.9)	5 (11.9)	19 (6.2)	0.44 (0.14,1.65)	0.167
Duration of α blockade (day)	41 (29, 60)	45.5 (35, 63)	41 (29, 59)	0.4	0.715
Phenoxybenzamine (mg/day)	30 (20, 30)	30 (20, 40)	30 (20, 30)	0.7	0.419
Hemodynamic variables before surgery					
SBP (mmHg)	136.4 ± 19.6	137.4 ± 17.9	136.3 ± 19.8	1.1	0.726
DBP (mmHg)	84.0 ± 13.1	85.5 ± 13.9	83.8 ± 13.0	1.7	0.436
HR (bpm)	80.3 ± 12.5	81.0 ± 14.4	80.2 ± 12.2	0.8	0.698
Surgical approach, n (%)				7.58 (1.96, 27.82)	<0.001
Laparoscopy	337(96.3)	36 (85.7)	301 (97.7)		
Converted to open laparotomy	13(3.7)	6 (14.3)	7 (2.3)		
Surgical duration (min)	117.3 ± 60.0	148.6 ± 98.4	113.0 ± 51.5	35.6	0.001
Estimated blood loss(ml)	100(50,300)	150(50, 600)	50 (50, 250)	4.7	0.001
RBC transfusion, n (%)	35(10)	9 (21.4)	26 (8.4)	2.96 (1.11, 7.20)	0.008
FFP transfusion, n (%)	17 (4.9)	7 (16.7)	10 (3.2)	5.96 (1.79, 18.48)	<0.001
Hemoglobin decrease (10 g.L^-1^ decrease)	1.8 ± 1.1	2.5 ± 1.6	1.7 ± 1.0	0.8	<0.001
Postoperative hypotension requiring vasopressor infusion, n (%)	206 (58.9)	32 (76.2)	174 (56.5)	2.46 (1.13, 5.81)	0.015
Length of stay (days)	14 (11, 20)	16 (12, 24)	14 (11, 19)	5.0	0.087

BMI, body mass index; ASA, American Society of Anesthesia; CCB, calcium channel blocker; SBP, systolic blood pressure; DBP, diastolic blood pressure; HR, heart rate; bpm, beats per minute; mmHg, millimeters of mercury; RBC, red blood cell; FFP, fresh frozen plasma; OR, odds ratio; CI, confidence interval; SD, standard deviation. Data are presented as mean ± SD, median (25th, 75th percentiles) or n (%),P-values from chi-square test, Student’s t-test, or Wilcoxon rank sum test, as appropriate. * means that the P-value cannot be calculated.

### Incidence of myocardial injury and other complications

The outcomes up to hospital discharge are presented in **Table 2**. No patient died in hospital. Myocardial injury was the most common cardiovascular complication, which occurred in 42/350 patients (12.0% [95% confidence interval (CI): 9.0%–15.9%]). Only 1/42 patients (2.4% [95% CI: 0.0%–2.0%]) fulfilled the fourth definition of myocardial infarction, and 97.6% patients with myocardial injury did not experience ischemic symptoms or significant electrocardiogram changes. Another postoperative cardiovascular adverse event was new atrial fibrillation (0.6% [95% CI: 0.1%–2.3%]). Acute kidney injury occurred in 18/350 patients (5.1% [95% CI: 3.3%–8.0%]). Pulmonary infection and surgical site infection occurred in 20 patients without elevated troponin I levels (5.7%).

**Table 2:** Outcome events up to hospital discharge following laparoscopic adrenalectomy for pheochromocytoma.

Outcome	n = 350 n (%)	95% CI (%)	Clavien-Dindo classification
In-hospital death	0 (0.0)		—
Myocardial injury*	41 (11.7)	8.7 to 15.5	Grade I
AKI	18 (5.1)	3.3 to 8.0	Grade I
SSI	8 (2.3)	1.1 to 4.5	Grade I
New atrial fibrillation	2 (0.6)	0.1 to 2.3	Grade II
Pulmonary infection	12 (3.4)	2.0 to 6.0	Grade II
Myocardial infarction	1 (0.3)	0.0 to 2.0	Grade IIIa

AKI, acute kidney injury; SSI, surgical site infection. CI, confidence interval. The data are presented as n (%). *Myocardial injury occurred in 42/350 patients (12.0% [95% confidence interval (CI): 9.0%–15.9%]), including myocardial infarction and no pulmonary infection or surgical site infection occurred in patients with myocardial injury.

### Summary of dichotomized intraoperative hemodynamics by myocardial injury

The incidence of transient tachycardia and hypertension was high in all patients, with approximately 90% patients having tachycardia (HR >100 bpm) and one-third having severe hypertension (SBP >210 mmHg). Furthermore, the incidence of hypertension and tachycardia was higher in the myocardial injury group and the difference increased progressively with higher BP and HR values. The dichotomized intraoperative hemodynamics in both groups are presented in **Supplementary Table S1**.

### Multivariable analysis of risk factors associated with myocardial injury

**Figure 2:**
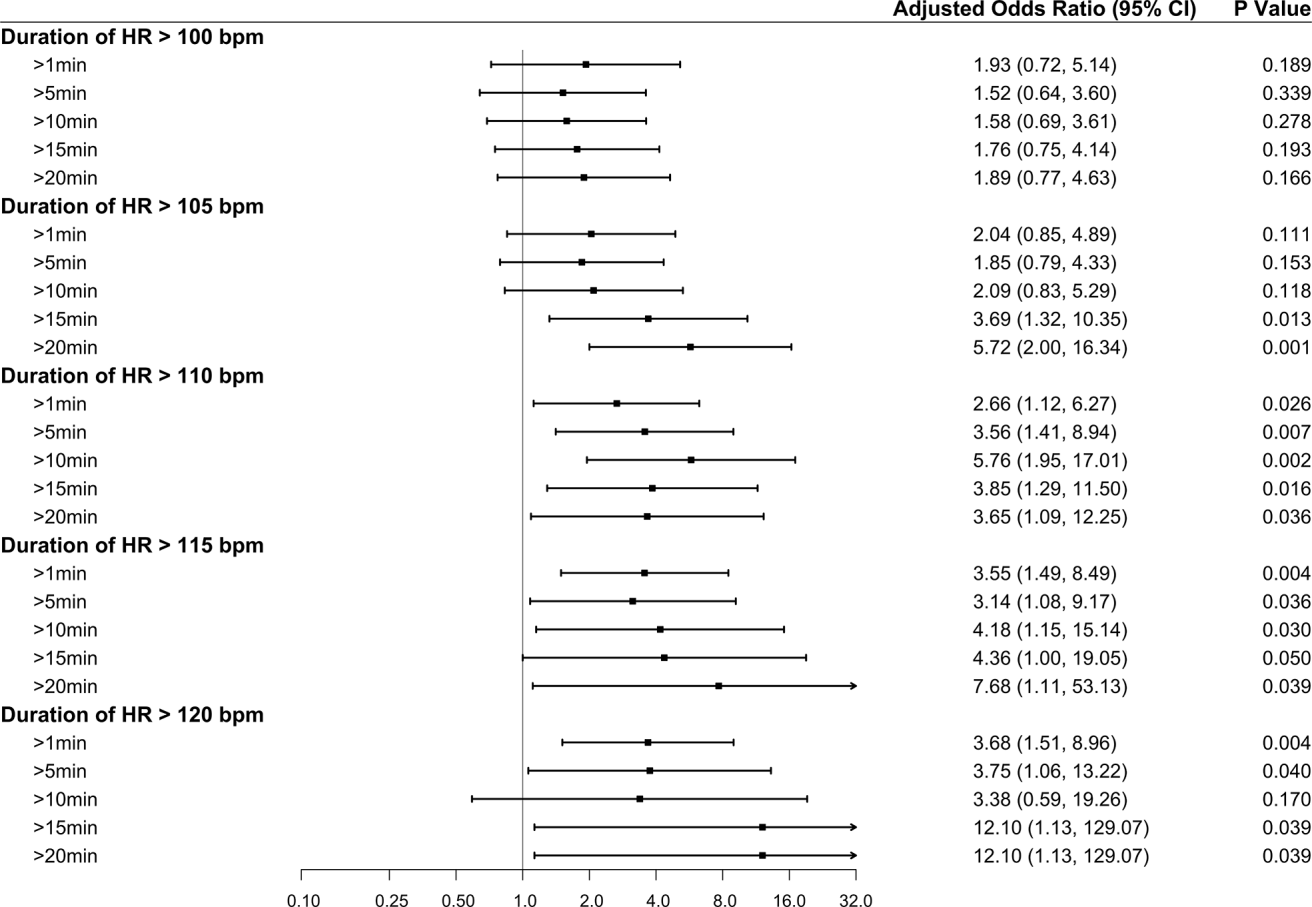
Forest plot summarizing the multivariable logistic regression models for the highest intraoperative heart rate and highest intraoperative systolic blood pressure. The highest intraoperative heart rate was dichotomized according to the thresholds of >100, >105, >110, >115, and >120 bpm, with ≤100, ≤105, ≤110, ≤115, and ≤120 bpm as the reference categories, respectively. The highest intraoperative systolic blood pressure was dichotomized according to the thresholds of >160, >180, >200, >210, and >220 mmHg, with ≤160, ≤180, ≤200, ≤210, and ≤220 mmHg as the reference categories, respectively. The x-axis represents odds ratios, and the error bars represent 95% confidence intervals. The full multivariable models are presented in **Supplementary Tables S3** and **S5–S13**.

In the multivariable analysis without intraoperative hemodynamic variables, previous ischemic heart disease or stroke and perioperative decrease in hemoglobin level were significantly associated with an increased risk of myocardial injury (**Table 3**, Model 1). In the multivariable analysis in Model 2, the highest intraoperative HR (>115 bpm) was another risk factor associated with myocardial injury (adjusted odds ratio [aOR], 2.83 [95% CI: 1.21–6.63]; *P*<0.017). In the multivariable analysis in Model 3, in addition to the above three risk factors, severe hypertension (SBP >210 mmHg) was associated with an increased risk of myocardial injury (aOR, 2.38 [95% CI: 1.004–5.66]; *P*=0.049). There was a trend toward an increased risk of myocardial injury as the highest intraoperative HR and SBP values increased (**Figure 2**). The full multivariable models are presented in **Supplementary Tables S2–S13**.

**Table 3:** Multivariable analysis models of risk factors associated with myocardial injury after laparoscopic adrenalectomy for pheochromocytoma.

	Model 1	Model 2	Model 3	Model 4
Factors	Adjusted OR (95% CI)	Adjusted OR(95% CI)	Adjusted OR (95% CI)	Adjusted OR (95% CI)
Previous ischemic heart disease or stroke, n (%)	6.06 (1.80–20.41)^$^	5.35 (1.55, 18.45)^$^	5.04 (1.40–18.08)*	3.98 (1.11– 14.32)*
Hemoglobin drop (g.dL^-1^ decrease)	1.70 (1.14– 2.54)*	1.76 (1.16– 2.66)^$^	1.74 (1.15– 2.64)^$^	1.79 (1.17– 2.73)^$^
HR > 115 bpm		2.83 (1.21– 6.63)*	2.55 (1.06– 6.12)*	
SBP > 210 mmHg			2.38 (1.00–5.66)*	
HR > 115 bpm over 1 min				2.67 (1.08–6.60)*
SBP > 210 mmHg over 1 min				3.78 (1.47–9.73)^$^

SBP, systolic blood pressure; HR, heart rate; bpm, beats per minute; mmHg, millimeters of mercury; OR, odds ratio; CI, confidence interval. * means P<0.05, ^$^ means P<0.01.

### Multivariable analysis of the cumulative time of dichotomized intraoperative HR and SBP values associated with myocardial injury

**Figure 3:**
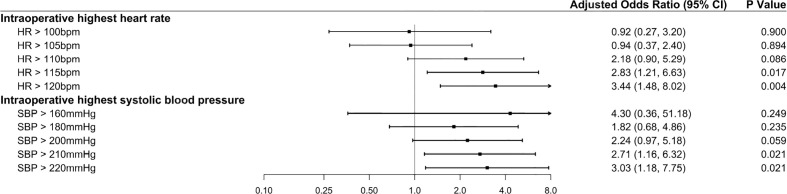
Forest plot summarizing the multivariable logistic regression models for the duration of elevated intraoperative heart rate. The duration of elevated intraoperative heart rate was stratified into five quartiles: cumulative time >1, >5, >10, >15, and >20 minutes. The reference category included patients with a “normal” heart rate. For example, in the analysis of the duration of elevated intraoperative heart rate >100 bpm, the reference category included patients with a heart rate ≤100 bpm. The x-axis represents odds ratios, and the error bars represent 95% confidence intervals.

In addition to the increased risk of myocardial injury due to an absolute HR increase, as the cumulative time increased, the risk of myocardial injury also gradually increased (**Figure 3**). Besides, the aOR appeared to be higher for tachycardia (HR >115 bpm) and hypertension (SBP >210 mmHg) persisting for >1 minute than for the dichotomized HR (HR >115 bpm) and SBP (SBP >210 mmHg) values (**Table 3**, Model 4). Based on tachycardia (HR >115 bpm) lasting for 1 minute, the higher the intraoperative SBP (SBP >200, >210, and >220 mmHg) persisting for 1 minute, the greater the risk of myocardial injury (aOR, 3.44 [95% CI: 3.78–3.92]). The full multivariable models are presented in **Supplementary Tables S14–S16**.

## Discussion

To our knowledge, this is the first study to determine the incidence and risk factors for myocardial injury after laparoscopic adrenalectomy for pheochromocytoma. Previous ischemic heart disease or stroke, severe intraoperative hypertension (SBP >210 mmHg), tachycardia (HR >115 bpm), and perioperative decrease in hemoglobin level were associated with postoperative myocardial injury.

The incidence of myocardial injury (12.0%) in our study was higher than that in previously reported non-cardiac abdominal surgeries (18, 30).However, only one patient was diagnosed with non-ST elevation myocardial infarction(NSTMI). In our opinion, a considerable proportion of patients with myocardial infarction (mainly type 2 myocardial infarction) may be underestimated in this study. According to the fourth universal definition of myocardial infarction (27), type 2 myocardial infarction is due to a mismatch between the oxygen supply and demand. This universal definition allows for distinction between type 2 myocardial infarction and myocardial injury based on the presence or absence of symptoms and signs of myocardial ischemia. It was likely that a higher proportion of patients, who suffered a myocardial injury, experienced ischemic ECG changes at some point in their postoperative course. However, these changes were likely frequently missed as the patients did not experience ischemic symptoms or the symptoms were hidden by opioids used in the ICU as a trigger for obtaining an ECG/ECHO during the period of ischemia. Therefore, it is not appropriate to dismiss episodes of myocardial injury as a mere bystander phenomenon of no clinical consequence.

In this study, we observed that previous ischemic heart disease or stroke were significantly associated with postoperative myocardial injury, consistent with many proposed clinical cardiac risk assessment tools (31–35). The patients in our study were generally younger than those of previous studies (32–34), with a mean age of approximately 45 years. Although the prevalence of previous ischemic heart disease or stroke in our study was relatively low (7.7%), the occurrence of adverse cardiovascular or cerebrovascular events indicated that the patients may had experienced abnormal hemodynamic fluctuations before surgery, particularly severe hypertension and tachycardia caused by catecholamine release.

Preoperative risk factors, such as patient characteristics and comorbidities, are stronger predictors of adverse cardiovascular outcomes than intraoperative blood pressure (22). Nevertheless, blood pressure is crucial because it can largely be controlled, unlike patient characteristics. In this retrospective cohort study, we found that severe intraoperative hypertension (SBP >210 mmHg) increased the risk of myocardial injury. Moreover, the risk of severe hypertension gradually increased with longer duration (SBP >210 mmHg persisting for >1 minute: aOR, 3.78 [95% CI: 1.47–9.73]), even close to the risk associated with previous ischemic heart disease or stroke (aOR, 3.98 [95% CI: 1.11–14.32]). Endotracheal intubation, insufflation of the abdomen with carbon dioxide for laparoscopy, and tumor manipulation may provoke instant catecholamine release with the potential for hypertensive crisis and tachycardia (8). Tachycardia increases myocardial oxygen demand and decreases diastolic filling time, which is believed to be the main reason for cardiac supply-demand mismatch during surgery. At present the degree and duration of tachycardia that promote myocardial injury are controversial. An intraoperative HR >100 bpm was not associated with myocardial injury in our study, comparable to the result in nearly 3,000 noncardiac surgical patients at the Cleveland Clinic (23). However, we noticed that the injury was apparent when the intraoperative HR exceeded 115 bpm (aOR, 2.55 [95% CI: 1.06–6.12]). Furthermore, the threshold for tachycardia gradually decreased as the cumulative duration increased in our study, consistent with the results of previous observational studies (24, 36).

A perioperative decrease in hemoglobin level reduced myocardial oxygen supply, which was associated with myocardial injury in our study. Massive intraoperative hemorrhage is usually the main reason for the significant decrease in perioperative hemoglobin level. In the POISE (PeriOperative ISchemic Evaluation) trial, severe bleeding (requiring ≥2 units of RBC) was a significant predictor of perioperative myocardial infarction (aOR, 3.62 [95% CI: 2.07–6.36]), which was also confirmed by the subsequent POISE-2 trial (33). However, intraoperative hemorrhage in our study was not significant (median [interquartile range]) (100 [50–300] mL). We thought that excessive intraoperative fluid infusion may lead to a dilutional decrease in hemoglobin level after surgery in this population in addition to intraoperative hemorrhage. Although the amount of blood loss and the transfused proportion of blood products in patients with myocardial injury were relatively higher, there were no statistical difference between the two groups in multivariable logistic regression analysis.

The current study included a relatively large sample size for rare tumors. However, there are some limitations. First, myocardial injury was defined by the peak troponin I level, which may underestimate the occurrence of myocardial injury compared with fourth- and fifth-generation high-sensitivity troponin T (16). Second, the retrospective nature of the study made it impossible to determine all postoperative complications, because monitoring for such complications was at the discretion of the treating physician. Besides, we did not perform post-discharge follow-up. Therefore, the effects of myocardial injury on longer-term outcomes remain unknown. Finally, our analysis was restricted to the highest values of intraoperative SBP and HR, which does not imply that lower blood pressure and bradycardia are necessarily safe. To assess the risk of hypotension and bradycardia, a similar analysis based on the lowest BP and HR values would be required.

In conclusion, we identified the incidence and perioperative risk factors for myocardial injury after laparoscopic adrenalectomy for pheochromocytoma. Perioperative monitoring of troponin I should be performed before surgery and during the first 2 days for this high-risk operation. Our results suggest that extra attention should be paid to patients with preexisting ischemic heart disease or stroke, severe intraoperative hypertension and tachycardia, and a significant perioperative decrease in hemoglobin levels. Further studies are required to confirm our results and explore the predictors of myocardial injury in this population.

Each multivariable model was adjusted for potentially confounding factors: male sex, the American Society of Anesthesiology (ASA) physical status, smoking history, alcohol use, diabetes, hypertension, previous ischemic heart disease or stroke, previous congestive heart failure, preoperative 24-h urinary catecholamine elevation (none, epinephrine elevated, norepinephrine elevated, dopamine elevated),tumor location, preoperative medications (α blockade only,α blockade + calcium channel blocker (CCB), α blockade + β blockade, α blockade + β blockade + CCB), surgical approach, red blood cells transfusion, fresh frozen plasma transfusion, postoperative hypotension requiring vasopressors were considered as categorical variables, and age, body mass index, preoperative creatine, multiple of the normal reference upper limit value (24-h urinary epinephrine, 24-h urinary norepinephrine, 24-h urinary dopamine), maximum tumor diameter, duration of *α* blockade (day), phenoxybenzamine (mg/day), hemodynamic variables the day preceding surgery(arterial systolic blood pressure, diastolic blood pressure, heart rate), surgical duration (min), blood loss (mL), and hemoglobin drop were considered as constant variables in the multivariable models. * P<0.05, ^$^ P<0.01. Full multivariable models are presented in the Supplemental Digital Content (**Tables S2–S4**, **S15**).

## Data availability statement

The raw data supporting the conclusions of this article will be made available by the authors, without undue reservation.

## Author contributions

LL contributed to the study design, data collection, statistical analysis, and writing of the original draft. QS contributed to data collection and review and editing. CY and LP contributed to the study design. YZ contributed to the statistical analysis and review and editing. LX and YH contributed to the conception, study design, interpretation of the data, and review and editing. All authors read and approved the final manuscript. All authors contributed to the article and approved the submitted version.

## Funding

This research did not receive any specific grant from funding agencies in the public, commercial, or not-for-profit sectors.

## Acknowledgments

The authors thank Dr. Penghao Liu of Peking Union Medical College, Chinese Academy of Medical Sciences, Beijing, China, for his help with figure preparation.

## Conflict of interest

The authors declare that the research was conducted in the absence of any commercial or financial relationships that could be construed as a potential conflict of interest.

## Publisher’s note

All claims expressed in this article are solely those of the authors and do not necessarily represent those of their affiliated organizations, or those of the publisher, the editors and the reviewers. Any product that may be evaluated in this article, or claim that may be made by its manufacturer, is not guaranteed or endorsed by the publisher.

## Abbreviations

aOR, adjusted odds ratio; CI, confidence interval; DBP, diastolic blood pressure; HR, heart rate; SBP, systolic blood pressure.
