# Supplementary material for: Incidence and risk factors for myocardial injury after laparoscopic adrenalectomy for pheochromocytoma: A retrospective cohort study
Source: Front Oncol. 2022 Sep 12;12:979994. doi: 10.3389/fonc.2022.979994 (PMC9511041; doi:10.3389/fonc.2022.979994)
Supplement: Supplementary file 1 [file DataSheet_1.docx]

**Supplemental Table S1.** Summary of dichotomized intraoperative heart rate and systolic blood pressure by postoperative myocardial injury.

| Factors | Total  (n = 350) | | Myocardial injury  (n = 42) | No Myocardial injury  (n = 308) | Unadjusted OR (95% CI) | *P* value | Adjusted OR (95% CI) | *P** value |
| --- | --- | --- | --- | --- | --- | --- | --- | --- |
| Intraoperative highest Heart Rate |  |  | |  |  |  |  |  |
| HR > 100bpm | 287(82%) | 38 (90.5%) | | 249 (80.8%) | 2.25(0.77,6.55) | 0.137 | 0.92 (0.27, 3.20) | 0.900 |
| HR > 105bpm | 242(69.1%) | 33 (78.6%) | | 209 (67.9%) | 1.74(0.80,3.77) | 0.163 | 0.94 (0.37, 2.40) | 0.894 |
| HR > 110bpm | 200(57.1%) | 32 (76.2%) | | 168 (54.5%) | 2.67(1.27,5.62) | 0.010 | 2.18 (0.90, 5.29) | 0.086 |
| HR > 115bpm | 157(44.9%) | 29 (69.0%) | | 128 (41.6%) | 3.14(1.57,6.27) | 0.001 | 2.83 (1.21, 6.63) | 0.017 |
| HR > 120bpm | 112(32%) | 24 (57.1%) | | 88 (28.6%) | 3.33(1.72,6.44) | <0.001 | 3.44 (1.48, 8.02) | 0.004 |
| Intraoperative highest Systolic Blood Pressure |  |  | |  |  |  |  |  |
| SBP > 160 mmHg | 317(90.6%) | 41 (97.6%) | | 276 (89.6%) | 4.75(0.63,35.73) | 0.130 | 4.30(0.36,51.18) | 0.249 |
| SBP > 180 mmHg | 234(66.9%) | 33 (78.6%) | | 201 (65.3%) | 1.95(0.90,4.23) | 0.090 | 1.82(0.68,4.86) | 0.235 |
| SBP > 200 mmHg | 149(42.6%) | 24 (57.1%) | | 125 (40.6%) | 1.95(1.02,3.75) | 0.044 | 2.24(0.97,5.18) | 0.059 |
| SBP > 210 mmHg | 107(30.6%) | 19 (45.2%) | | 88 (28.6%) | 2.07(1.07,3.98) | 0.030 | 2.71 (1.07,3.98） | 0.021 |
| SBP > 220 mmHg | 63(18%) | 13 (31.0%) | | 50 (16.2%) | 2.31(1.12,4.76) | 0.023 | 3.03(1.18,7.75) | 0.021 |

HR, heart rate; SBP, systolic blood pressure; bpm, beat per minute; mmHg, millimeter of mercury; OR, odds ratio; CI, continuous integration. Highest intraoperative heart rate was dichotomized according to thresholds of >100 bpm, >105 bpm, >110 bpm, >115 bpm and 120 bpm, with heart rate ≤100 bpm, ≤105 bpm, ≤110 bpm, ≤115 bpm, ≤120 bpm as the reference category respectively. Highest intraoperative systolic blood pressure was dichotomized according to thresholds of >160 mmHg, >180 mmHg, >200 mmHg, >210 mmHg and >220 mmHg, with systolic blood pressure ≤160 mmHg, ≤180 mmHg, ≤200 mmHg, ≤210 mmHg and ≤220 mmHg as the reference category respectively. Data are represented as n (%). *P* value from univariable logistic regression, *P* *value from multivariable logistic regression;

**Supplemental Table S2.** Multivariable logistic regression model without intraoperative haemodynamic variables.

| Factors | Myocardial injury  (n = 42) | No myocardial injury  (n = 308) | | Adjusted OR (95% CI) | *P* value | |  |
| --- | --- | --- | --- | --- | --- | --- | --- |
| Age, yr | 44.4 ± 14.5 | 44.6 ± 13.3 | | 0.98(0.94, 1.02) | 0.313 | |  |
| Male, n (%) | 22 (52.4%) | 139 (45.1%) | | 0.57 (0.16, 1.94) | 0.365 | |  |
| BMI, kg/m^2^ | 23.3 ± 3.1 | 23.9 ± 3.3 | | 0.91 (0.80, 1.04) | 0.181 | |  |
| ASA physical status, n (%) |  |  | | 1.66 (0.79, 3.52) | 0.182 | |  |
| 1 | 0 (0.0) | 9 (2.9%) | |  |  | |  |
| 2 | 15 (35.7%) | 127 (41.2%) | |  |  | |  |
| 3 | 27 (64.3%) | 172 (55.8%) | |  |  | |  |
| Smoking history, n (%) | 12 (28.6%) | 51 (16.6%) | | 1.42 (0.43, 4.75） | 0.568 | |  |
| Alcohol use, n (%) | 10 (23.8%) | 50 (16.2%) | | 2.09 (0.61,7.20) | 0.242 | |  |
| Diabetes, n (%) | 9 (21.4%) | 55 (17.9%) | | 1.02 (0.61, 1.71) | 0.934 | |  |
| Hypertension, n (%) | 20 (47.6%) | 137 (44.5%) | | 0.63 (0.34, 1.18) | 0.148 | |  |
| Previous ischemic heart disease or stroke, n (%) | 8 (19.0%) | 19 (6.2%) | | 6.06 (1.80, 20.41) | 0.004 | |  |
| Congestive heart failure, n (%) | 4 (9.5%) | 17 (5.5%) | | 1.79 (0.43, 7.49) | 0.427 | |  |
| Preoperative creatine (µmol/L) | 70.4 ± 17.4 | 67.9 ± 14.5 | | 1.01 (0.98, 1.05) | 0.515 | |  |
| Preoperative 24-h urinary catecholamine elevated level |  |  | | 0.97(0.57,1.63) | 0.902 | |  |
| None | 74 (24.0%) | 11 (26.2%) | |  |  | |  |
| E elevated | 71 (23.1%) | 10 (23.8%) | |  |  | |  |
| NE elevated | 159 (51.6%) | 20 (47.6%) | |  |  | |  |
| DA elevated | 4 (1.3%) | 1 (2.4%) | |  |  | |  |
| Multiple of the normal reference upper limit value |  |  |  | |  |  | |
| 24-h urinary NE^*^ | 6.9 ± 18.7 | 4.8 ± 14.4 | | 0.99 (0.94, 1.03) | 0.498 | |  |
| 24-h urinary E^*^ | 7.7 ± 13.6 | 6.1 ± 9.6 | | 1.01 (0.99, 1.04) | 0.299 | |  |
| 24-h urinary D^*^ | 0.9 ± 0.6 | 1.0 ± 1.3 | | 0.74 (0.37, 1.49) | 0.396 | |  |
| Tumor location, n (%) |  |  | | 0.38 (0.07, 2.06) | 0.260 | |  |
| Unilateral | 39 (92.9%) | 283 (91.9%) | |  |  | |  |
| Bilateral | 3 (7.1%) | 25 (8.1%) | |  |  | |  |
| Maximum tumor diameter (cm) | 5.2 ± 2.3 | 4.7 ± 1.8 | | 0.95 (0.75, 1.21) | 0.687 | |  |
| Preoperative medications, n (%) |  |  | | 1.15 (0.75,1.75) | 0.520 | |  |
| *α* blockade only | 24 (57.1%) | 207 (67.2%) | |  |  | |  |
| *α* blockade +CCB | 8 (19.0%) | 24 (7.8%) | |  |  | |  |
| *α* blockade+*β* blockade | 5 (11.9%) | 58 (18.8%) | |  |  | |  |
| *α* blockade+*β* blockade+CCB | 5 (11.9%) | 19 (6.2%) | |  |  | |  |
| Duration of *α* blockade (day) | 45.5 (35, 63) | 41 (29, 59) | | 1.00 (0.99, 1.01) | 0.779 | |  |
| Phenoxybenzamine (mg/day) | 30 (20, 40) | 30 (20, 30) | | 1.00 (0.98, 1.02) | 0.831 | |  |
| Hemodynamic variables day before surgery |  |  | |  |  | |  |
| SBP (mmHg) | 137.4 ± 17.9 | 136.3 ± 19.8 | | 1.00 (0.96, 1.03) | 0.919 | |  |
| DBP (mmHg) | 85.5 ± 13.9 | 83.8 ± 13.0 | | 1.01 (0.96, 1.06) | 0.716 | |  |
| HR (bpm) | 81.0 ± 14.4 | 80.2 ± 12.2 | | 1.00 (0.97, 1.04) | 0.850 | |  |
| Surgical approach, n (%) |  |  | | 2.41 (0.74, 7.82) | 0.142 | |  |
| laparoscopy | 36 (85.7%) | 301 (97.7%) | |  |  | |  |
| Converted to open laparotomy | 6 (14.3%) | 7 (2.3%) | |  |  | |  |
| Surgical duration (min) | 148.6 ± 98.4 | 113.0 ± 51.5 | | 1.00 (0.99, 1.01) | 0.835 | |  |
| Blood loss(ml) | 150(50, 600) | 50 (50, 250) | | 1.00 (1.00, 1.00) | 0.213 | |  |
| RBC transfusion, n (%) | 9 (21.4%) | 26 (8.4%) | | 0.28 (0.04, 2.26) | 0.233 | |  |
| FFP transfusion, n (%) | 7 (16.7%) | 10 (3.2%) | | 3.53 (0.25, 49.50) | 0.348 | |  |
| Hemoglobin drop (10g.L^-1^ decrease) | 2.5 ± 1.6 | 1.7 ± 1.0 | | 1.70 (1.14, 2.54) | 0.010 | |  |
| Postoperative hypotension requiring vasopressors, n (%) | 32 (76.2%) | 174 (56.5%) | | 1.89 (0.79, 4.48) | 0.152 | |  |

BMI, body mass index; ASA, American Society of Anesthesia; NE, norepinephrine; E, epinephrine; DA, dopamine; CCB, calcium channel blockers; SBP, systolic blood pressure; DBP, diastolic blood pressure; HR, heart rate; bpm, beat per minute; mmHg, millimeter of mercury; RBC, red blood cell; FFP, fresh frozen plasma; LOS, length of stay. Data are represented as mean ± SD, median [25th, 75th percentiles] or n (%). OR,odds ratio; CI, confidence interval.^*^multiple of the normal reference upper limit value. P value from multivariable logistic regression;

**Supplemental Table S3.** Multivariable logistic regression model for intraoperative highest heart rate over 115bpm.

| Factors | Myocardial injury  (n = 42) | No myocardial injury  (n = 308) | | | Adjusted OR (95% CI) | | *P* value |
| --- | --- | --- | --- | --- | --- | --- | --- |
| HR > 115 bpm | 29(69.0%) | 128(41.6%) | | | 2.83(1.21,6.63) | | 0.017 |
| Age, yr | 44.4 ± 14.5 | 44.6 ± 13.3 | | | 0.99(0.95, 1.03) | | 0.467 |
| Male, n (%) | 22 (52.4%) | 139 (45.1%) | | | 0.56 (0.16, 1.96) | | 0.364 |
| BMI, kg/m^2^ | 23.3 ± 3.1 | 23.9 ± 3.3 | | | 0.92 (0.81, 1.05) | | 0.244 |
| ASA physical status, n (%) |  |  | | | 1.51 (0.71, 3.22) | | 0.283 |
| 1 | 0 (0.0) | 9 (2.9%) | | |  | |  |
| 2 | 15 (35.7%) | 127 (41.2%) | | |  | |  |
| 3 | 27 (64.3%) | 172 (55.8%) | | |  | |  |
| Smoking history, n (%) | 12 (28.6%) | 51 (16.6%) | | | 1.55 (0.45,5.34） | | 0.484 |
| Alcohol use, n (%) | 10 (23.8%) | 50 (16.2%) | | | 2.06(0.57,7.41) | | 0.270 |
| Diabetes, n (%) | 9 (21.4%) | 55 (17.9%) | | | 1.02 (0.60, 1.72) | | 0.951 |
| Hypertension, n (%) | 20 (47.6%) | 137 (44.5%) | | | 0.58 (0.30, 1.09) | | 0.092 |
| Previous ischemic heart disease or stroke, n (%) | 8 (19.0%) | 19 (6.2%) | | | 5.35 (1.55, 18.45) | | 0.008 |
| Congestive heart failure, n (%) | 4 (9.5%) | 17 (5.5%) | | | 2.07 (0.49, 8.79) | | 0.324 |
| Preoperative creatine (µmol/L) | 70.4 ± 17.4 | 67.9 ± 14.5 | | | 1.01 (0.97, 1.05) | | 0.575 |
| Preoperative 24-h urinary catecholamine elevated level |  |  | | | 1.03(0.61,1.74) | | 0.926 |
| None | 74 (24.0%) | 11 (26.2%) | | |  | |  |
| E elevated | 71 (23.1%) | 10 (23.8%) | | |  | |  |
| NE elevated | 159 (51.6%) | 20 (47.6%) | | |  | |  |
| DA elevated | 4 (1.3%) | 1 (2.4%) | | |  | |  |
| Multiple of the normal reference upper limit value |  |  |  |  | |  |  |
| 24-h urinary NE^*^ | 6.9 ± 18.7 | 4.8 ± 14.4 | | | 0.98 (0.93, 1.02) | | 0.338 |
| 24-h urinary E^*^ | 7.7 ± 13.6 | 6.1 ± 9.6 | | | 1.01 (0.99, 1.04) | | 0.302 |
| 24-h urinary D^*^ | 0.9 ± 0.6 | 1.0 ± 1.3 | | | 0.73 (0.35, 1.50) | | 0.385 |
| Tumor location, n (%) |  |  | | | 0.34 (0.06, 1.86) | | 0.212 |
| Unilateral | 39 (92.9%) | 283 (91.9%) | | |  | |  |
| Bilateral | 3 (7.1%) | 25 (8.1%) | | |  | |  |
| Maximum tumor diameter (cm) | 5.2 ± 2.3 | 4.7 ± 1.8 | | | 0.92 (0.73, 1.17) | | 0.509 |
| Preoperative medications, n (%) |  |  | | | 1.23 (0.80,1.89) | | 0.343 |
| *α* blockade only | 24 (57.1%) | 207 (67.2%) | | |  | |  |
| *α* blockade +CCB | 8 (19.0%) | 24 (7.8%) | | |  | |  |
| *α* blockade+*β* blockade | 5 (11.9%) | 58 (18.8%) | | |  | |  |
| *α* blockade+*β* blockade+CCB | 5 (11.9%) | 19 (6.2%) | | |  | |  |
| Duration of *α* blockade (day) | 45.5 (35, 63) | 41 (29, 59) | | | 1.00 (0.98, 1.01) | | 0.619 |
| Phenoxybenzamine (mg/day) | 30 (20, 40) | 30 (20, 30) | | | 1.23 (0.80, 1.89) | | 0.343 |
| Hemodynamic variables day before surgery |  |  | | |  | |  |
| SBP (mmHg) | 137.4 ± 17.9 | 136.3 ± 19.8 | | | 0.99 (0.96, 1.03) | | 0.765 |
| DBP (mmHg) | 85.5 ± 13.9 | 83.8 ± 13.0 | | | 1.01 (0.97, 1.06) | | 0.556 |
| HR (bpm) | 81.0 ± 14.4 | 80.2 ± 12.2 | | | 0.99 (0.96, 1.03) | | 0.767 |
| Surgical approach, n (%) |  |  | | | 2.68 (0.84, 8.53) | | 0.096 |
| laparoscopy | 36 (85.7%) | 301 (97.7%) | | |  | |  |
| Converted to open laparotomy | 6 (14.3%) | 7 (2.3%) | | |  | |  |
| Surgical duration (min) | 148.6 ± 98.4 | 113.0 ± 51.5 | | | 1.00 (0.99, 1.01) | | 0.691 |
| Blood loss(ml) | 150(50, 600) | 50 (50, 250) | | | 1.00 (1.00, 1.00) | | 0.235 |
| RBC transfusion, n (%) | 9 (21.4%) | 26 (8.4%) | | | 0.31 (0.04, 2.49) | | 0.270 |
| FFP transfusion, n (%) | 7 (16.7%) | 10 (3.2%) | | | 3.13 (0.21, 45.75) | | 0.405 |
| Hemoglobin drop (10g.L^-1^ decrease) | 2.5 ± 1.6 | 1.7 ± 1.0 | | | 1.76 (1.16, 2.66) | | 0.007 |
| Postoperative hypotension requiring vasopressors, n (%) | 32 (76.2%) | 174 (56.5%) | | | 1.75 (0.72, 4.23) | | 0.218 |

BMI, body mass index; ASA, American Society of Anesthesia; NE, norepinephrine; E, epinephrine; DA, dopamine; CCB, calcium channel blockers; SBP, systolic blood pressure; DBP, diastolic blood pressure; HR, heart rate; bpm, beat per minute; mmHg, millimeter of mercury; RBC, red blood cell; FFP, fresh frozen plasma; LOS, length of stay. Data are represented as mean ± SD, median [25th, 75th percentiles] or n (%).OR,odds ratio; CI, confidence interval.^*^multiple of the normal reference upper limit value. P value from multivariable logistic regression;

**Supplemental Table S4.** Multivariable logistic regression model for intraoperative highest heart rate over 115bpm and systolic blood pressure over 210 mmHg.

| Factors | Myocardial injury  (n = 42) | No myocardial injury  (n = 308) | | | Adjusted OR (95% CI) | | *P* value |
| --- | --- | --- | --- | --- | --- | --- | --- |
| SBP > 210 mmHg | 19(45.2%) | 88(28.6%) | | | 2.38(1.00,5.66) | | 0.049 |
| HR > 115 bpm | 29(69.0%) | 128(41.6%) | | | 2.55(1.06,6.12) | | 0.036 |
| Age, yr | 44.4 ± 14.5 | 44.6 ± 13.3 | | | 0.98(0.94, 1.02) | | 0.404 |
| Male, n (%) | 22 (52.4%) | 139 (45.1%) | | | 0.48 (0.13, 1.71) | | 0.257 |
| BMI, kg/m^2^ | 23.3 ± 3.1 | 23.9 ± 3.3 | | | 0.92 (0.80, 1.05) | | 0.203 |
| ASA physical status, n (%) |  |  | | | 1.54 (0.72, 3.33) | | 0.267 |
| 1 | 0 (0.0) | 9 (2.9%) | | |  | |  |
| 2 | 15 (35.7%) | 127 (41.2%) | | |  | |  |
| 3 | 27 (64.3%) | 172 (55.8%) | | |  | |  |
| Smoking history, n (%) | 12 (28.6%) | 51 (16.6%) | | | 1.76 (0.49,6.35） | | 0.386 |
| Alcohol use, n (%) | 10 (23.8%) | 50 (16.2%) | | | 2.07(0.56,7.75) | | 0.278 |
| Diabetes, n (%) | 9 (21.4%) | 55 (17.9%) | | | 1.03 (0.61, 1.75) | | 0.908 |
| Hypertension, n (%) | 20 (47.6%) | 137 (44.5%) | | | 0.53 (0.27, 1.03) | | 0.061 |
| Previous ischemic heart disease or stroke, n (%) | 8 (19.0%) | 19 (6.2%) | | | 5.04 (1.40, 18.08) | | 0.013 |
| Congestive heart failure, n (%) | 4 (9.5%) | 17 (5.5%) | | | 2.10 (0.49, 9.00) | | 0.316 |
| Preoperative creatine (µmol/L) | 70.4 ± 17.4 | 67.9 ± 14.5 | | | 1.01 (0.98, 1.05) | | 0.473 |
| Preoperative 24-h urinary catecholamine elevated level |  |  | | | 1.00(0.58,1.70) | | 0.989 |
| None | 74 (24.0%) | 11 (26.2%) | | |  | |  |
| E elevated | 71 (23.1%) | 10 (23.8%) | | |  | |  |
| NE elevated | 159 (51.6%) | 20 (47.6%) | | |  | |  |
| DA elevated | 4 (1.3%) | 1 (2.4%) | | |  | |  |
| Multiple of the normal reference upper limit value |  |  |  |  | |  |  |
| 24-h urinary NE^*^ | 6.9 ± 18.7 | 4.8 ± 14.4 | | | 0.97 (0.93, 1.02) | | 0.294 |
| 24-h urinary E^*^ | 7.7 ± 13.6 | 6.1 ± 9.6 | | | 1.01 (0.99, 1.04) | | 0.312 |
| 24-h urinary D^*^ | 0.9 ± 0.6 | 1.0 ± 1.3 | | | 0.73 (0.35, 1.53) | | 0.408 |
| Tumor location, n (%) |  |  | | | 0.36 (0.06, 2.07) | | 0.249 |
| Unilateral | 39 (92.9%) | 283 (91.9%) | | |  | |  |
| Bilateral | 3 (7.1%) | 25 (8.1%) | | |  | |  |
| Maximum tumor diameter (cm) | 5.2 ± 2.3 | 4.7 ± 1.8 | | | 0.88 (0.69, 1.12) | | 0.312 |
| Preoperative medications, n (%) |  |  | | | 1.18 (0.77,1.84) | | 0.439 |
| *α* blockade only | 24 (57.1%) | 207 (67.2%) | | |  | |  |
| *α* blockade +CCB | 8 (19.0%) | 24 (7.8%) | | |  | |  |
| *α* blockade+*β* blockade | 5 (11.9%) | 58 (18.8%) | | |  | |  |
| *α* blockade+*β* blockade+CCB | 5 (11.9%) | 19 (6.2%) | | |  | |  |
| Duration of *α* blockade (day) | 45.5 (35, 63) | 41 (29, 59) | | | 1.00 (0.98, 1.01) | | 0.655 |
| Phenoxybenzamine (mg/day) | 30 (20, 40) | 30 (20, 30) | | | 1.00 (0.98, 1.03) | | 0.706 |
| Hemodynamic variables day before surgery |  |  | | |  | |  |
| SBP (mmHg) | 137.4 ± 17.9 | 136.3 ± 19.8 | | | 0.99 (0.96, 1.03) | | 0.684 |
| DBP (mmHg) | 85.5 ± 13.9 | 83.8 ± 13.0 | | | 1.01 (0.97, 1.06) | | 0.596 |
| HR (bpm) | 81.0 ± 14.4 | 80.2 ± 12.2 | | | 1.00(0.96, 1.03) | | 0.939 |
| Surgical approach, n (%) |  |  | | | 3.04 (0.93, 9.91) | | 0.065 |
| laparoscopy | 36 (85.7%) | 301 (97.7%) | | |  | |  |
| Converted to open laparotomy | 6 (14.3%) | 7 (2.3%) | | |  | |  |
| Surgical duration (min) | 148.6 ± 98.4 | 113.0 ± 51.5 | | | 1.00 (0.99, 1.01) | | 0.456 |
| Blood loss(ml) | 150(50, 600) | 50 (50, 250) | | | 1.00 (1.00, 1.00) | | 0.091 |
| RBC transfusion, n (%) | 9 (21.4%) | 26 (8.4%) | | | 0.25 (0.03, 2.24) | | 0.215 |
| FFP transfusion, n (%) | 7 (16.7%) | 10 (3.2%) | | | 2.70 (0.17, 43.09) | | 0.481 |
| Hemoglobin drop (10g.L^-1^ decrease) | 2.5 ± 1.6 | 1.7 ± 1.0 | | | 1.74 (1.15, 2.64) | | 0.008 |
| Postoperative hypotension requiring vasopressors, n (%) | 32 (76.2%) | 174 (56.5%) | | | 1.85 (0.75, 4.55) | | 0.181 |

BMI, body mass index; ASA, American Society of Anesthesia; NE, norepinephrine; E, epinephrine; DA, dopamine; CCB, calcium channel blockers; SBP, systolic blood pressure; DBP, diastolic blood pressure; HR, heart rate; bpm, beat per minute; mmHg, millimeter of mercury; RBC, red blood cell; FFP, fresh frozen plasma; LOS, length of stay. Data are represented as mean ± SD, median [25th, 75th percentiles] or n (%). OR,odds ratio; CI, confidence interval.^*^multiple of the normal reference upper limit value. P value from multivariable logistic regression;

**Supplemental Table S5.** Multivariable logistic regression model for intraoperative highest heart rate over 100bpm.

| Factors | Myocardial injury  (n = 42) | No myocardial injury  (n = 308) | | | Adjusted OR (95% CI) | | *P* value |
| --- | --- | --- | --- | --- | --- | --- | --- |
| HR > 100 bpm | 38(90.5%) | 249(80.8%) | | | 0.92(0.27,3.20) | | 0.900 |
| Age, yr | 44.4 ± 14.5 | 44.6 ± 13.3 | | | 0.98(0.94, 1.02) | | 0.310 |
| Male, n (%) | 22 (52.4%) | 139 (45.1%) | | | 0.56 (0.16, 1.94) | | 0.361 |
| BMI, kg/m^2^ | 23.3 ± 3.1 | 23.9 ± 3.3 | | | 0.91 (0.80, 1.04) | | 0.179 |
| ASA physical status, n (%) |  |  | | | 1.66 (0.79, 3.52) | | 0.180 |
| 1 | 0 (0.0) | 9 (2.9%) | | |  | |  |
| 2 | 15 (35.7%) | 127 (41.2%) | | |  | |  |
| 3 | 27 (64.3%) | 172 (55.8%) | | |  | |  |
| Smoking history, n (%) | 12 (28.6%) | 51 (16.6%) | | | 1.42 (0.42,4.73） | | 0.573 |
| Alcohol use, n (%) | 10 (23.8%) | 50 (16.2%) | | | 2.11(0.61,7.32) | | 0.239 |
| Diabetes, n (%) | 9 (21.4%) | 55 (17.9%) | | | 1.02 (0.61, 1.71) | | 0.943 |
| Hypertension, n (%) | 20 (47.6%) | 137 (44.5%) | | | 0.63 (0.34, 1.18) | | 0.148 |
| Previous ischemic heart disease or stroke, n (%) | 8 (19.0%) | 19 (6.2%) | | | 6.13 (1.79, 20.97) | | 0.004 |
| Congestive heart failure, n (%) | 4 (9.5%) | 17 (5.5%) | | | 1.80 (0.43, 7.62) | | 0.800 |
| Preoperative creatine (µmol/L) | 70.4 ± 17.4 | 67.9 ± 14.5 | | | 1.01 (0.98, 1.05) | | 0.660 |
| Preoperative 24-h urinary catecholamine elevated level |  |  | | | 0.97(0.57,1.64) | | 0.907 |
| None | 74 (24.0%) | 11 (26.2%) | | |  | |  |
| E elevated | 71 (23.1%) | 10 (23.8%) | | |  | |  |
| NE elevated | 159 (51.6%) | 20 (47.6%) | | |  | |  |
| DA elevated | 4 (1.3%) | 1 (2.4%) | | |  | |  |
| Multiple of the normal reference upper limit value |  |  |  |  | |  |  |
| 24-h urinary NE^*^ | 6.9 ± 18.7 | 4.8 ± 14.4 | | | 1.01 (0.99, 1.04) | | 0.296 |
| 24-h urinary E^*^ | 7.7 ± 13.6 | 6.1 ± 9.6 | | | 0.99 (0.94, 1.03) | | 0.499 |
| 24-h urinary D^*^ | 0.9 ± 0.6 | 1.0 ± 1.3 | | | 0.74 (0.37, 1.49) | | 0.397 |
| Tumor location, n (%) |  |  | | | 0.37 (0.07, 2.05) | | 0.258 |
| Unilateral | 39 (92.9%) | 283 (91.9%) | | |  | |  |
| Bilateral | 3 (7.1%) | 25 (8.1%) | | |  | |  |
| Maximum tumor diameter (cm) | 5.2 ± 2.3 | 4.7 ± 1.8 | | | 0.95 (0.75, 1.21) | | 0.692 |
| Preoperative medications, n (%) |  |  | | | 1.14 (0.75,1.75) | | 0.539 |
| *α* blockade only | 24 (57.1%) | 207 (67.2%) | | |  | |  |
| *α* blockade +CCB | 8 (19.0%) | 24 (7.8%) | | |  | |  |
| *α* blockade+*β* blockade | 5 (11.9%) | 58 (18.8%) | | |  | |  |
| *α* blockade+*β* blockade+CCB | 5 (11.9%) | 19 (6.2%) | | |  | |  |
| Duration of *α* blockade (day) | 45.5 (35, 63) | 41 (29, 59) | | | 1.00 (0.99, 1.01) | | 0.777 |
| Phenoxybenzamine (mg/day) | 30 (20, 40) | 30 (20, 30) | | | 1.00 (0.98, 1.02) | | 0.829 |
| Hemodynamic variables day before surgery |  |  | | |  | |  |
| SBP (mmHg) | 137.4 ± 17.9 | 136.3 ± 19.8 | | | 1.00 (0.96, 1.03) | | 0.914 |
| DBP (mmHg) | 85.5 ± 13.9 | 83.8 ± 13.0 | | | 1.01 (0.97, 1.06) | | 0.712 |
| HR (bpm) | 81.0 ± 14.4 | 80.2 ± 12.2 | | | 1.00 (0.97, 1.04) | | 0.837 |
| Surgical approach, n (%) |  |  | | | 2.41 (0.74, 7.82) | | 0.142 |
| laparoscopy | 36 (85.7%) | 301 (97.7%) | | |  | |  |
| Converted to open laparotomy | 6 (14.3%) | 7 (2.3%) | | |  | |  |
| Surgical duration (min) | 148.6 ± 98.4 | 113.0 ± 51.5 | | | 1.00 (0.99, 1.01) | | 0.848 |
| Blood loss(ml) | 150(50, 600) | 50 (50, 250) | | | 1.00 (1.00, 1.00) | | 0.213 |
| RBC transfusion, n (%) | 9 (21.4%) | 26 (8.4%) | | | 0.28 (0.03, 2.26) | | 0.232 |
| FFP transfusion, n (%) | 7 (16.7%) | 10 (3.2%) | | | 3.53 (0.25, 49.58) | | 0.349 |
| Hemoglobin drop (10g.L^-1^ decrease) | 2.5 ± 1.6 | 1.7 ± 1.0 | | | 1.70 (1.14, 2.54) | | 0.010 |
| Postoperative hypotension requiring vasopressors, n (%) | 32 (76.2%) | 174 (56.5%) | | | 1.89 (0.79, 4.52) | | 0.150 |

BMI, body mass index; ASA, American Society of Anesthesia; NE, norepinephrine; E, epinephrine; DA, dopamine; CCB, calcium channel blockers; SBP, systolic blood pressure; DBP, diastolic blood pressure; HR, heart rate; bpm, beat per minute; mmHg, millimeter of mercury; RBC, red blood cell; FFP, fresh frozen plasma; LOS, length of stay. Data are represented as mean ± SD, median [25th, 75th percentiles] or n (%). OR,odds ratio; CI, confidence interval.^*^multiple of the normal reference upper limit value. P value from multivariable logistic regression;

**Supplemental Table S6.** Multivariable logistic regression model for intraoperative highest heart rate over 105bpm.

| Factors | Myocardial injury  (n = 42) | No myocardial injury  (n = 308) | | | Adjusted OR (95% CI) | | *P* value |
| --- | --- | --- | --- | --- | --- | --- | --- |
| HR > 105 bpm | 33(78.5%) | 209(67.9%) | | | 0.94(0.37,2.40) | | 0.894 |
| Age, yr | 44.4 ± 14.5 | 44.6 ± 13.3 | | | 0.98(0.94, 1.02) | | 0.309 |
| Male, n (%) | 22 (52.4%) | 139 (45.1%) | | | 0.56 (0.16, 1.94) | | 0.361 |
| BMI, kg/m^2^ | 23.3 ± 3.1 | 23.9 ± 3.3 | | | 0.91 (0.80, 1.04) | | 0.179 |
| ASA physical status, n (%) |  |  | | | 1.67 (0.79, 3.55) | | 0.181 |
| 1 | 0 (0.0) | 9 (2.9%) | | |  | |  |
| 2 | 15 (35.7%) | 127 (41.2%) | | |  | |  |
| 3 | 27 (64.3%) | 172 (55.8%) | | |  | |  |
| Smoking history, n (%) | 12 (28.6%) | 51 (16.6%) | | | 1.42 (0.42,4.74） | | 0.569 |
| Alcohol use, n (%) | 10 (23.8%) | 50 (16.2%) | | | 2.09(0.61,7.20) | | 0.241 |
| Diabetes, n (%) | 9 (21.4%) | 55 (17.9%) | | | 1.02 (0.61, 1.71) | | 0.933 |
| Hypertension, n (%) | 20 (47.6%) | 137 (44.5%) | | | 0.63 (0.34, 1.18) | | 0.152 |
| Previous ischemic heart disease or stroke, n (%) | 8 (19.0%) | 19 (6.2%) | | | 6.09 (1.80, 20.63) | | 0.004 |
| Congestive heart failure, n (%) | 4 (9.5%) | 17 (5.5%) | | | 1.80 (0.43, 7.50) | | 0.429 |
| Preoperative creatine (µmol/L) | 70.4 ± 17.4 | 67.9 ± 14.5 | | | 1.01 (0.98, 1.05) | | 0.511 |
| Preoperative 24-h urinary catecholamine elevated level |  |  | | | 0.97(0.57,1.64) | | 0.907 |
| None | 74 (24.0%) | 11 (26.2%) | | |  | |  |
| E elevated | 71 (23.1%) | 10 (23.8%) | | |  | |  |
| NE elevated | 159 (51.6%) | 20 (47.6%) | | |  | |  |
| DA elevated | 4 (1.3%) | 1 (2.4%) | | |  | |  |
| Multiple of the normal reference upper limit value |  |  |  |  | |  |  |
| 24-h urinary NE^*^ | 6.9 ± 18.7 | 4.8 ± 14.4 | | | 0.99 (0.94, 1.03) | | 0.303 |
| 24-h urinary E^*^ | 7.7 ± 13.6 | 6.1 ± 9.6 | | | 1.01 (0.99, 1.04) | | 0.498 |
| 24-h urinary D^*^ | 0.9 ± 0.6 | 1.0 ± 1.3 | | | 0.74 (0.37, 1.49) | | 0.399 |
| Tumor location, n (%) |  |  | | | 0.37 (0.07, 2.06) | | 0.260 |
| Unilateral | 39 (92.9%) | 283 (91.9%) | | |  | |  |
| Bilateral | 3 (7.1%) | 25 (8.1%) | | |  | |  |
| Maximum tumor diameter (cm) | 5.2 ± 2.3 | 4.7 ± 1.8 | | | 0.95 (0.75, 1.21) | | 0.687 |
| Preoperative medications, n (%) |  |  | | | 1.14 (0.75,1.75) | | 0.535 |
| *α* blockade only | 24 (57.1%) | 207 (67.2%) | | |  | |  |
| *α* blockade +CCB | 8 (19.0%) | 24 (7.8%) | | |  | |  |
| *α* blockade+*β* blockade | 5 (11.9%) | 58 (18.8%) | | |  | |  |
| *α* blockade+*β* blockade+CCB | 5 (11.9%) | 19 (6.2%) | | |  | |  |
| Duration of *α* blockade (day) | 45.5 (35, 63) | 41 (29, 59) | | | 1.00 (0.99, 1.01) | | 0.778 |
| Phenoxybenzamine (mg/day) | 30 (20, 40) | 30 (20, 30) | | | 1.00 (0.98, 1.02) | | 0.825 |
| Hemodynamic variables day before surgery |  |  | | |  | |  |
| SBP (mmHg) | 137.4 ± 17.9 | 136.3 ± 19.8 | | | 1.00 (0.96, 1.03) | | 0.917 |
| DBP (mmHg) | 85.5 ± 13.9 | 83.8 ± 13.0 | | | 1.01 (0.96, 1.06) | | 0.714 |
| HR (bpm) | 81.0 ± 14.4 | 80.2 ± 12.2 | | | 1.00 (0.97, 1.04) | | 0.840 |
| Surgical approach, n (%) |  |  | | | 2.41 (0.74, 7.80) | | 0.143 |
| laparoscopy | 36 (85.7%) | 301 (97.7%) | | |  | |  |
| Converted to open laparotomy | 6 (14.3%) | 7 (2.3%) | | |  | |  |
| Surgical duration (min) | 148.6 ± 98.4 | 113.0 ± 51.5 | | | 1.00 (0.99, 1.01) | | 0.857 |
| Blood loss(ml) | 150(50, 600) | 50 (50, 250) | | | 1.00 (1.00, 1.00) | | 0.215 |
| RBC transfusion, n (%) | 9 (21.4%) | 26 (8.4%) | | | 0.28 (0.04, 2.25) | | 0.232 |
| FFP transfusion, n (%) | 7 (16.7%) | 10 (3.2%) | | | 3.56 (0.25, 50.09) | | 0.346 |
| Hemoglobin drop (10g.L^-1^ decrease) | 2.5 ± 1.6 | 1.7 ± 1.0 | | | 1.70 (1.14, 2.54) | | 0.010 |
| Postoperative hypotension requiring vasopressors, n (%) | 32 (76.2%) | 174 (56.5%) | | | 1.90 (0.79, 4.52) | | 0.151 |

BMI, body mass index; ASA, American Society of Anesthesia; NE, norepinephrine; E, epinephrine; DA, dopamine; CCB, calcium channel blockers; SBP, systolic blood pressure; DBP, diastolic blood pressure; HR, heart rate; bpm, beat per minute; mmHg, millimeter of mercury; RBC, red blood cell; FFP, fresh frozen plasma; LOS, length of stay. Data are represented as mean ± SD, median [25th, 75th percentiles] or n (%). OR,odds ratio; CI, confidence interval.^*^multiple of the normal reference upper limit value. P value from multivariable logistic regression;

**Supplemental Table S7.** Multivariable logistic regression model for intraoperative highest heart rate over 110bpm.

| Factors | Myocardial injury  (n = 42) | No myocardial injury  (n = 308) | | | Adjusted OR (95% CI) | | *P* value |
| --- | --- | --- | --- | --- | --- | --- | --- |
| HR > 110 bpm | 32(76.2%) | 168(54.5%) | | | 2.18(0.90,5.29) | | 0.086 |
| Age, yr | 44.4 ± 14.5 | 44.6 ± 13.3 | | | 0.98(0.94, 1.02) | | 0.401 |
| Male, n (%) | 22 (52.4%) | 139 (45.1%) | | | 0.56 (0.16, 1.94) | | 0.361 |
| BMI, kg/m^2^ | 23.3 ± 3.1 | 23.9 ± 3.3 | | | 0.93 (0.81, 1.06) | | 0.275 |
| ASA physical status, n (%) |  |  | | | 1.52 (0.72, 3.24) | | 0.272 |
| 1 | 0 (0.0) | 9 (2.9%) | | |  | |  |
| 2 | 15 (35.7%) | 127 (41.2%) | | |  | |  |
| 3 | 27 (64.3%) | 172 (55.8%) | | |  | |  |
| Smoking history, n (%) | 12 (28.6%) | 51 (16.6%) | | | 1.52 (0.45,5.09） | | 0.500 |
| Alcohol use, n (%) | 10 (23.8%) | 50 (16.2%) | | | 2.01(0.58,7.07) | | 0.273 |
| Diabetes, n (%) | 9 (21.4%) | 55 (17.9%) | | | 1.05 (0.62, 1.77) | | 0.856 |
| Hypertension, n (%) | 20 (47.6%) | 137 (44.5%) | | | 0.63 (0.34, 1.18) | | 0.119 |
| Previous ischemic heart disease or stroke, n (%) | 8 (19.0%) | 19 (6.2%) | | | 5.55 (1.63, 18.89) | | 0.006 |
| Congestive heart failure, n (%) | 4 (9.5%) | 17 (5.5%) | | | 1.90 (0.45, 8.05) | | 0.382 |
| Preoperative creatine (µmol/L) | 70.4 ± 17.4 | 67.9 ± 14.5 | | | 1.01 (0.98, 1.05) | | 0.439 |
| Preoperative 24-h urinary catecholamine elevated level |  |  | | | 0.98(0.58,1.66) | | 0.955 |
| None | 74 (24.0%) | 11 (26.2%) | | |  | |  |
| E elevated | 71 (23.1%) | 10 (23.8%) | | |  | |  |
| NE elevated | 159 (51.6%) | 20 (47.6%) | | |  | |  |
| DA elevated | 4 (1.3%) | 1 (2.4%) | | |  | |  |
| Multiple of the normal reference upper limit value |  |  |  |  | |  |  |
| 24-h urinary NE^*^ | 6.9 ± 18.7 | 4.8 ± 14.4 | | | 0.98 (0.94, 1.03) | | 0.307 |
| 24-h urinary E^*^ | 7.7 ± 13.6 | 6.1 ± 9.6 | | | 1.01 (0.99, 1.04) | | 0.423 |
| 24-h urinary D^*^ | 0.9 ± 0.6 | 1.0 ± 1.3 | | | 0.73 (0.36, 1.50) | | 0.393 |
| Tumor location, n (%) |  |  | | | 0.34 (0.06, 1.86) | | 0.212 |
| Unilateral | 39 (92.9%) | 283 (91.9%) | | |  | |  |
| Bilateral | 3 (7.1%) | 25 (8.1%) | | |  | |  |
| Maximum tumor diameter (cm) | 5.2 ± 2.3 | 4.7 ± 1.8 | | | 0.94 (0.74, 1.19) | | 0.595 |
| Preoperative medications, n (%) |  |  | | | 1.24 (0.81,1.91) | | 0.324 |
| *α* blockade only | 24 (57.1%) | 207 (67.2%) | | |  | |  |
| *α* blockade +CCB | 8 (19.0%) | 24 (7.8%) | | |  | |  |
| *α* blockade+*β* blockade | 5 (11.9%) | 58 (18.8%) | | |  | |  |
| *α* blockade+*β* blockade+CCB | 5 (11.9%) | 19 (6.2%) | | |  | |  |
| Duration of *α* blockade (day) | 45.5 (35, 63) | 41 (29, 59) | | | 1.00 (0.99, 1.01) | | 0.699 |
| Phenoxybenzamine (mg/day) | 30 (20, 40) | 30 (20, 30) | | | 1.24 (0.81, 1.91) | | 0.918 |
| Hemodynamic variables day before surgery |  |  | | |  | |  |
| SBP (mmHg) | 137.4 ± 17.9 | 136.3 ± 19.8 | | | 1.00 (0.96, 1.03) | | 0.867 |
| DBP (mmHg) | 85.5 ± 13.9 | 83.8 ± 13.0 | | | 1.01 (0.96, 1.06) | | 0.688 |
| HR (bpm) | 81.0 ± 14.4 | 80.2 ± 12.2 | | | 1.00 (0.97, 1.03) | | 0.989 |
| Surgical approach, n (%) |  |  | | | 2.50 (0.78, 8.04) | | 0.125 |
| laparoscopy | 36 (85.7%) | 301 (97.7%) | | |  | |  |
| Converted to open laparotomy | 6 (14.3%) | 7 (2.3%) | | |  | |  |
| Surgical duration (min) | 148.6 ± 98.4 | 113.0 ± 51.5 | | | 1.00 (0.99, 1.01) | | 0.688 |
| Blood loss(ml) | 150(50, 600) | 50 (50, 250) | | | 1.00 (1.00, 1.00) | | 0.222 |
| RBC transfusion, n (%) | 9 (21.4%) | 26 (8.4%) | | | 0.33 (0.04, 2.67) | | 0.299 |
| FFP transfusion, n (%) | 7 (16.7%) | 10 (3.2%) | | | 2.93 (0.21, 41.92) | | 0.428 |
| Hemoglobin drop (10g.L^-1^ decrease) | 2.5 ± 1.6 | 1.7 ± 1.0 | | | 1.73 (1.15, 2.60) | | 0.008 |
| Postoperative hypotension requiring vasopressors, n (%) | 32 (76.2%) | 174 (56.5%) | | | 1.80 (0.75, 4.34) | | 0.188 |

BMI, body mass index; ASA, American Society of Anesthesia; NE, norepinephrine; E, epinephrine; DA, dopamine; CCB, calcium channel blockers; SBP, systolic blood pressure; DBP, diastolic blood pressure; HR, heart rate; bpm, beat per minute; mmHg, millimeter of mercury; RBC, red blood cell; FFP, fresh frozen plasma; LOS, length of stay. Data are represented as mean ± SD, median [25th, 75th percentiles] or n (%).OR,odds ratio; CI, confidence interval.^*^multiple of the normal reference upper limit value. P value from multivariable logistic regression;

**Supplemental Table S8.** Multivariable logistic regression model for intraoperative highest heart rate over 120bpm.

| Factors | Myocardial injury  (n = 42) | No myocardial injury  (n = 308) | | | Adjusted OR (95% CI) | | *P* value |
| --- | --- | --- | --- | --- | --- | --- | --- |
| HR > 120 bpm | 24(57.1%) | 88(28.6%) | | | 3.44(1.48,8.02) | | 0.004 |
| Age, yr | 44.4 ± 14.5 | 44.6 ± 13.3 | | | 0.99(0.95, 1.03) | | 0.527 |
| Male, n (%) | 22 (52.4%) | 139 (45.1%) | | | 0.52 (0.15, 1.80) | | 0.302 |
| BMI, kg/m^2^ | 23.3 ± 3.1 | 23.9 ± 3.3 | | | 0.92 (0.81, 1.05) | | 0.241 |
| ASA physical status, n (%) |  |  | | | 1.44 (0.68, 3.08) | | 0.342 |
| 1 | 0 (0.0) | 9 (2.9%) | | |  | |  |
| 2 | 15 (35.7%) | 127 (41.2%) | | |  | |  |
| 3 | 27 (64.3%) | 172 (55.8%) | | |  | |  |
| Smoking history, n (%) | 12 (28.6%) | 51 (16.6%) | | | 1.60 (0.47,5.48） | | 0.453 |
| Alcohol use, n (%) | 10 (23.8%) | 50 (16.2%) | | | 1.84(0.52,6.57) | | 0.347 |
| Diabetes, n (%) | 9 (21.4%) | 55 (17.9%) | | | 1.02 (0.60, 1.72) | | 0.911 |
| Hypertension, n (%) | 20 (47.6%) | 137 (44.5%) | | | 0.55 (0.29, 1.06) | | 0.075 |
| Previous ischemic heart disease or stroke, n (%) | 8 (19.0%) | 19 (6.2%) | | | 5.44 (1.58, 18.69) | | 0.007 |
| Congestive heart failure, n (%) | 4 (9.5%) | 17 (5.5%) | | | 2.07 (0.48, 8.95) | | 0.329 |
| Preoperative creatine (µmol/L) | 70.4 ± 17.4 | 67.9 ± 14.5 | | | 1.01 (0.98, 1.05) | | 0.457 |
| Preoperative 24-h urinary catecholamine elevated level |  |  | | | 1.00(0.59,1.69) | | 0.990 |
| None | 74 (24.0%) | 11 (26.2%) | | |  | |  |
| E elevated | 71 (23.1%) | 10 (23.8%) | | |  | |  |
| NE elevated | 159 (51.6%) | 20 (47.6%) | | |  | |  |
| DA elevated | 4 (1.3%) | 1 (2.4%) | | |  | |  |
| Multiple of the normal reference upper limit value |  |  |  |  | |  |  |
| 24-h urinary NE^*^ | 6.9 ± 18.7 | 4.8 ± 14.4 | | | 0.98 (0.93, 1.02) | | 0.333 |
| 24-h urinary E^*^ | 7.7 ± 13.6 | 6.1 ± 9.6 | | | 1.01 (0.99, 1.04) | | 0.353 |
| 24-h urinary D^*^ | 0.9 ± 0.6 | 1.0 ± 1.3 | | | 0.75 (0.38, 1.46) | | 0.391 |
| Tumor location, n (%) |  |  | | | 0.34 (0.06, 1.94) | | 0.223 |
| Unilateral | 39 (92.9%) | 283 (91.9%) | | |  | |  |
| Bilateral | 3 (7.1%) | 25 (8.1%) | | |  | |  |
| Maximum tumor diameter (cm) | 5.2 ± 2.3 | 4.7 ± 1.8 | | | 0.90 (0.71, 1.14) | | 0.401 |
| Preoperative medications, n (%) |  |  | | | 1.25 (0.81,1.91) | | 0.314 |
| *α* blockade only | 24 (57.1%) | 207 (67.2%) | | |  | |  |
| *α* blockade +CCB | 8 (19.0%) | 24 (7.8%) | | |  | |  |
| *α* blockade+*β* blockade | 5 (11.9%) | 58 (18.8%) | | |  | |  |
| *α* blockade+*β* blockade+CCB | 5 (11.9%) | 19 (6.2%) | | |  | |  |
| Duration of *α* blockade (day) | 45.5 (35, 63) | 41 (29, 59) | | | 1.00 (0.98, 1.01) | | 0.680 |
| Phenoxybenzamine (mg/day) | 30 (20, 40) | 30 (20, 30) | | | 1.00 (0.98, 1.03) | | 0.729 |
| Hemodynamic variables day before surgery |  |  | | |  | |  |
| SBP (mmHg) | 137.4 ± 17.9 | 136.3 ± 19.8 | | | 0.99 (0.96, 1.03) | | 0.673 |
| DBP (mmHg) | 85.5 ± 13.9 | 83.8 ± 13.0 | | | 1.02 (0.97, 1.07) | | 0.548 |
| HR (bpm) | 81.0 ± 14.4 | 80.2 ± 12.2 | | | 1.00 (0.96, 1.03) | | 0.794 |
| Surgical approach, n (%) |  |  | | | 2.55 (0.81, 8.02) | | 0.109 |
| laparoscopy | 36 (85.7%) | 301 (97.7%) | | |  | |  |
| Converted to open laparotomy | 6 (14.3%) | 7 (2.3%) | | |  | |  |
| Surgical duration (min) | 148.6 ± 98.4 | 113.0 ± 51.5 | | | 1.00 (0.99, 1.01) | | 0.665 |
| Blood loss(ml) | 150(50, 600) | 50 (50, 250) | | | 1.00 (1.00, 1.00) | | 0.218 |
| RBC transfusion, n (%) | 9 (21.4%) | 26 (8.4%) | | | 0.27 (0.04, 2.16) | | 0.218 |
| FFP transfusion, n (%) | 7 (16.7%) | 10 (3.2%) | | | 3.34 (0.23, 48.18) | | 0.375 |
| Hemoglobin drop (10g.L^-1^ decrease) | 2.5 ± 1.6 | 1.7 ± 1.0 | | | 1.76 (1.14, 2.57) | | 0.009 |
| Postoperative hypotension requiring vasopressors, n (%) | 32 (76.2%) | 174 (56.5%) | | | 1.81 (0.74, 4.43) | | 0.196 |

BMI, body mass index; ASA, American Society of Anesthesia; NE, norepinephrine; E, epinephrine; DA, dopamine; CCB, calcium channel blockers; SBP, systolic blood pressure; DBP, diastolic blood pressure; HR, heart rate; bpm, beat per minute; mmHg, millimeter of mercury; RBC, red blood cell; FFP, fresh frozen plasma; LOS, length of stay. Data are represented as mean ± SD, median [25th, 75th percentiles] or n (%).OR,odds ratio; CI, confidence interval.^*^multiple of the normal reference upper limit value. P value from multivariable logistic regression;

**Supplemental Table S9.** Multivariable logistic regression model for intraoperative highest systolic blood pressure over 160 mmHg.

| Factors | Myocardial injury  (n = 42) | No myocardial injury  (n = 308) | | | Adjusted OR (95% CI) | | *P* value |
| --- | --- | --- | --- | --- | --- | --- | --- |
| SBP > 160 mmHg | 41(97.6%) | 276(89.6%) | | | 4.29(0.36,51.18) | | 0.249 |
| Age, yr | 44.4 ± 14.5 | 44.6 ± 13.3 | | | 0.99(0.95, 1.03) | | 0.392 |
| Male, n (%) | 22 (52.4%) | 139 (45.1%) | | | 0.52 (0.15, 1.80) | | 0.331 |
| BMI, kg/m^2^ | 23.3 ± 3.1 | 23.9 ± 3.3 | | | 0.92 (0.81, 1.05) | | 0.242 |
| ASA physical status, n (%) |  |  | | | 1.44 (0.68, 3.08) | | 0.214 |
| 1 | 0 (0.0) | 9 (2.9%) | | |  | |  |
| 2 | 15 (35.7%) | 127 (41.2%) | | |  | |  |
| 3 | 27 (64.3%) | 172 (55.8%) | | |  | |  |
| Smoking history, n (%) | 12 (28.6%) | 51 (16.6%) | | | 1.60 (0.47,5.48） | | 0.552 |
| Alcohol use, n (%) | 10 (23.8%) | 50 (16.2%) | | | 1.84(0.52,6.57) | | 0.267 |
| Diabetes, n (%) | 9 (21.4%) | 55 (17.9%) | | | 1.02 (0.60, 1.72) | | 0.976 |
| Hypertension, n (%) | 20 (47.6%) | 137 (44.5%) | | | 0.55 (0.29, 1.06) | | 0.134 |
| Previous ischemic heart disease or stroke, n (%) | 8 (19.0%) | 19 (6.2%) | | | 5.44 (1.58, 18.69) | | 0.005 |
| Congestive heart failure, n (%) | 4 (9.5%) | 17 (5.5%) | | | 2.07 (0.48, 8.95) | | 0.402 |
| Preoperative creatine (µmol/L) | 70.4 ± 17.4 | 67.9 ± 14.5 | | | 1.01 (0.98, 1.05) | | 0.476 |
| Preoperative 24-h urinary catecholamine elevated level |  |  | | | 1.00(0.59,1.69) | | 0.824 |
| None | 74 (24.0%) | 11 (26.2%) | | |  | |  |
| E elevated | 71 (23.1%) | 10 (23.8%) | | |  | |  |
| NE elevated | 159 (51.6%) | 20 (47.6%) | | |  | |  |
| DA elevated | 4 (1.3%) | 1 (2.4%) | | |  | |  |
| Multiple of the normal reference upper limit value |  |  |  |  | |  |  |
| 24-h urinary NE^*^ | 6.9 ± 18.7 | 4.8 ± 14.4 | | | 0.99 (0.95, 1.03) | | 0.547 |
| 24-h urinary E^*^ | 7.7 ± 13.6 | 6.1 ± 9.6 | | | 1.01 (0.99, 1.04) | | 0.315 |
| 24-h urinary D^*^ | 0.9 ± 0.6 | 1.0 ± 1.3 | | | 0.73 (0.37, 1.46) | | 0.372 |
| Tumor location, n (%) |  |  | | | 0.36 (0.07, 1.96) | | 0.238 |
| Unilateral | 39 (92.9%) | 283 (91.9%) | | |  | |  |
| Bilateral | 3 (7.1%) | 25 (8.1%) | | |  | |  |
| Maximum tumor diameter (cm) | 5.2 ± 2.3 | 4.7 ± 1.8 | | | 0.96 (0.76, 1.21) | | 0.711 |
| Preoperative medications, n (%) |  |  | | | 1.14 (0.75,1.73) | | 0.550 |
| *α* blockade only | 24 (57.1%) | 207 (67.2%) | | |  | |  |
| *α* blockade +CCB | 8 (19.0%) | 24 (7.8%) | | |  | |  |
| *α* blockade+*β* blockade | 5 (11.9%) | 58 (18.8%) | | |  | |  |
| *α* blockade+*β* blockade+CCB | 5 (11.9%) | 19 (6.2%) | | |  | |  |
| Duration of *α* blockade (day) | 45.5 (35, 63) | 41 (29, 59) | | | 1.00 (0.98, 1.01) | | 0.723 |
| Phenoxybenzamine (mg/day) | 30 (20, 40) | 30 (20, 30) | | | 1.00 (0.98, 1.02) | | 0.860 |
| Hemodynamic variables day before surgery |  |  | | |  | |  |
| SBP (mmHg) | 137.4 ± 17.9 | 136.3 ± 19.8 | | | 0.99 (0.96, 1.03) | | 0.775 |
| DBP (mmHg) | 85.5 ± 13.9 | 83.8 ± 13.0 | | | 1.01 (0.96, 1.06) | | 0.701 |
| HR (bpm) | 81.0 ± 14.4 | 80.2 ± 12.2 | | | 1.00 (0.97, 1.04) | | 0.796 |
| Surgical approach, n (%) |  |  | | | 2.91 (0.85, 9,95) | | 0.088 |
| laparoscopy | 36 (85.7%) | 301 (97.7%) | | |  | |  |
| Converted to open laparotomy | 6 (14.3%) | 7 (2.3%) | | |  | |  |
| Surgical duration (min) | 148.6 ± 98.4 | 113.0 ± 51.5 | | | 1.00 (0.99, 1.01) | | 0.713 |
| Blood loss(ml) | 150(50, 600) | 50 (50, 250) | | | 1.00 (1.00, 1.00) | | 0.205 |
| RBC transfusion, n (%) | 9 (21.4%) | 26 (8.4%) | | | 0.33 (0.04, 2.49) | | 0.280 |
| FFP transfusion, n (%) | 7 (16.7%) | 10 (3.2%) | | | 2.77 (0.20, 38.54) | | 0.448 |
| Hemoglobin drop (10g.L^-1^ decrease) | 2.5 ± 1.6 | 1.7 ± 1.0 | | | 1.73 (1.15, 2.60) | | 0.008 |
| Postoperative hypotension requiring vasopressors, n (%) | 32 (76.2%) | 174 (56.5%) | | | 1.75 (0.74, 4.18) | | 0.205 |

BMI, body mass index; ASA, American Society of Anesthesia; NE, norepinephrine; E, epinephrine; DA, dopamine; CCB, calcium channel blockers; SBP, systolic blood pressure; DBP, diastolic blood pressure; HR, heart rate; bpm, beat per minute; mmHg, millimeter of mercury; RBC, red blood cell; FFP, fresh frozen plasma; LOS, length of stay. Data are represented as mean ± SD, median [25th, 75th percentiles] or n (%). OR,odds ratio; CI, confidence interval.^*^multiple of the normal reference upper limit value. P value from multivariable logistic regression;

**Supplemental Table S10.** Multivariable logistic regression model for intraoperative highest systolic blood pressure over 180 mmHg.

| Factors | Myocardial injury  (n = 42) | No myocardial injury  (n = 308) | | | Adjusted OR (95% CI) | | *P* value |
| --- | --- | --- | --- | --- | --- | --- | --- |
| SBP > 180 mmHg | 33(78.6%) | 201(65.2%) | | | 1.82(0.67,4.86) | | 0.235 |
| Age, yr | 44.4 ± 14.5 | 44.6 ± 13.3 | | | 0.98(0.94, 1.02) | | 0.328 |
| Male, n (%) | 22 (52.4%) | 139 (45.1%) | | | 0.53 (0.15, 1.83) | | 0.318 |
| BMI, kg/m^2^ | 23.3 ± 3.1 | 23.9 ± 3.3 | | | 0.91 (0.80, 1.04) | | 0.184 |
| ASA physical status, n (%) |  |  | | | 1.59 (0.76, 3.34) | | 0.222 |
| 1 | 0 (0.0) | 9 (2.9%) | | |  | |  |
| 2 | 15 (35.7%) | 127 (41.2%) | | |  | |  |
| 3 | 27 (64.3%) | 172 (55.8%) | | |  | |  |
| Smoking history, n (%) | 12 (28.6%) | 51 (16.6%) | | | 1.49 (0.45,4.96） | | 0.511 |
| Alcohol use, n (%) | 10 (23.8%) | 50 (16.2%) | | | 2.00 (0.59,6.81) | | 0.267 |
| Diabetes, n (%) | 9 (21.4%) | 55 (17.9%) | | | 1.01 (0.61, 1.70) | | 0.957 |
| Hypertension, n (%) | 20 (47.6%) | 137 (44.5%) | | | 0.63 (0.34, 1.17) | | 0.143 |
| Previous ischemic heart disease or stroke, n (%) | 8 (19.0%) | 19 (6.2%) | | | 5.67 (1.65, 19.47) | | 0.006 |
| Congestive heart failure, n (%) | 4 (9.5%) | 17 (5.5%) | | | 1.76 (0.42, 7.38) | | 0.441 |
| Preoperative creatine (µmol/L) | 70.4 ± 17.4 | 67.9 ± 14.5 | | | 1.01 (0.98, 1.05) | | 0.461 |
| Preoperative 24-h urinary catecholamine elevated level |  |  | | | 0.97 (0.58,1.65) | | 0.923 |
| None | 74 (24.0%) | 11 (26.2%) | | |  | |  |
| E elevated | 71 (23.1%) | 10 (23.8%) | | |  | |  |
| NE elevated | 159 (51.6%) | 20 (47.6%) | | |  | |  |
| DA elevated | 4 (1.3%) | 1 (2.4%) | | |  | |  |
| Multiple of the normal reference upper limit value |  |  |  |  | |  |  |
| 24-h urinary NE^*^ | 6.9 ± 18.7 | 4.8 ± 14.4 | | | 0.98 (0.94, 1.03) | | 0.442 |
| 24-h urinary E^*^ | 7.7 ± 13.6 | 6.1 ± 9.6 | | | 1.01 (0.99, 1.04) | | 0.343 |
| 24-h urinary D^*^ | 0.9 ± 0.6 | 1.0 ± 1.3 | | | 0.74 (0.38, 1.45) | | 0.385 |
| Tumor location, n (%) |  |  | | | 0.40 (0.07, 2.19) | | 0.290 |
| Unilateral | 39 (92.9%) | 283 (91.9%) | | |  | |  |
| Bilateral | 3 (7.1%) | 25 (8.1%) | | |  | |  |
| Maximum tumor diameter (cm) | 5.2 ± 2.3 | 4.7 ± 1.8 | | | 0.95 (0.75, 1.20) | | 0.669 |
| Preoperative medications, n (%) |  |  | | | 1.14 (0.74,1.74) | | 0.554 |
| *α* blockade only | 24 (57.1%) | 207 (67.2%) | | |  | |  |
| *α* blockade +CCB | 8 (19.0%) | 24 (7.8%) | | |  | |  |
| *α* blockade+*β* blockade | 5 (11.9%) | 58 (18.8%) | | |  | |  |
| *α* blockade+*β* blockade+CCB | 5 (11.9%) | 19 (6.2%) | | |  | |  |
| Duration of *α* blockade (day) | 45.5 (35, 63) | 41 (29, 59) | | | 1.00 (0.99, 1.01) | | 0.728 |
| Phenoxybenzamine (mg/day) | 30 (20, 40) | 30 (20, 30) | | | 1.00 (0.98, 1.02) | | 0.892 |
| Hemodynamic variables day before surgery |  |  | | |  | |  |
| SBP (mmHg) | 137.4 ± 17.9 | 136.3 ± 19.8 | | | 0.99 (0.96, 1.03) | | 0.777 |
| DBP (mmHg) | 85.5 ± 13.9 | 83.8 ± 13.0 | | | 1.01 (0.96, 1.06) | | 0.742 |
| HR (bpm) | 81.0 ± 14.4 | 80.2 ± 12.2 | | | 1.00 (0.97, 1.04) | | 0.766 |
| Surgical approach, n (%) |  |  | | | 2.59 (0.81, 8.33) | | 0.110 |
| laparoscopy | 36 (85.7%) | 301 (97.7%) | | |  | |  |
| Converted to open laparotomy | 6 (14.3%) | 7 (2.3%) | | |  | |  |
| Surgical duration (min) | 148.6 ± 98.4 | 113.0 ± 51.5 | | | 1.00 (0.99, 1.01) | | 0.740 |
| Blood loss(ml) | 150(50, 600) | 50 (50, 250) | | | 1.00 (1.00, 1.00) | | 0.217 |
| RBC transfusion, n (%) | 9 (21.4%) | 26 (8.4%) | | | 0.30 (0.04, 2.33) | | 0.247 |
| FFP transfusion, n (%) | 7 (16.7%) | 10 (3.2%) | | | 3.45 (0.24, 48.66) | | 0.360 |
| Hemoglobin drop (10g.L^-1^ decrease) | 2.5 ± 1.6 | 1.7 ± 1.0 | | | 1.73 (1.15, 2.60) | | 0.012 |
| Postoperative hypotension requiring vasopressors, n (%) | 32 (76.2%) | 174 (56.5%) | | | 1.86 (0.78, 4.44) | | 0.165 |

BMI, body mass index; ASA, American Society of Anesthesia; NE, norepinephrine; E, epinephrine; DA, dopamine; CCB, calcium channel blockers; SBP, systolic blood pressure; DBP, diastolic blood pressure; HR, heart rate; bpm, beat per minute; mmHg, millimeter of mercury; RBC, red blood cell; FFP, fresh frozen plasma; LOS, length of stay. Data are represented as mean ± SD, median [25th, 75th percentiles] or n (%). OR,odds ratio; CI, confidence interval.^*^multiple of the normal reference upper limit value. P value from multivariable logistic regression;

**Supplemental Table S11.** Multivariable logistic regression model for intraoperative highest systolic blood pressure over 200 mmHg.

| Factors | Myocardial injury  (n = 42) | No myocardial injury  (n = 308) | | | Adjusted OR (95% CI) | | *P* value |
| --- | --- | --- | --- | --- | --- | --- | --- |
| SBP > 200 mmHg | 24(57.1%) | 125(40.6%) | | | 2.24(0.97,5.18) | | 0.059 |
| Age, yr | 44.4 ± 14.5 | 44.6 ± 13.3 | | | 0.98(0.94, 1.02) | | 0.328 |
| Male, n (%) | 22 (52.4%) | 139 (45.1%) | | | 0.52 (0.15, 1.80) | | 0.304 |
| BMI, kg/m^2^ | 23.3 ± 3.1 | 23.9 ± 3.3 | | | 0.92 (0.80, 1.05) | | 0.205 |
| ASA physical status, n (%) |  |  | | | 1.63 (0.77, 3.46) | | 0.202 |
| 1 | 0 (0.0) | 9 (2.9%) | | |  | |  |
| 2 | 15 (35.7%) | 127 (41.2%) | | |  | |  |
| 3 | 27 (64.3%) | 172 (55.8%) | | |  | |  |
| Smoking history, n (%) | 12 (28.6%) | 51 (16.6%) | | | 1.55 (0.45,5.31） | | 0.485 |
| Alcohol use, n (%) | 10 (23.8%) | 50 (16.2%) | | | 1.86 (0.53,6.54) | | 0.330 |
| Diabetes, n (%) | 9 (21.4%) | 55 (17.9%) | | | 1.00 (0.59, 1.68) | | 0.999 |
| Hypertension, n (%) | 20 (47.6%) | 137 (44.5%) | | | 0.63 (0.33, 1.18) | | 0.148 |
| Previous ischemic heart disease or stroke, n (%) | 8 (19.0%) | 19 (6.2%) | | | 6.07 (1.75, 21.05) | | 0.004 |
| Congestive heart failure, n (%) | 4 (9.5%) | 17 (5.5%) | | | 1.59 (0.37, 6.82) | | 0.532 |
| Preoperative creatine (µmol/L) | 70.4 ± 17.4 | 67.9 ± 14.5 | | | 1.01 (0.98, 1.05) | | 0.471 |
| Preoperative 24-h urinary catecholamine elevated level |  |  | | | 0.97 (0.57,1.65) | | 0.919 |
| None | 74 (24.0%) | 11 (26.2%) | | |  | |  |
| E elevated | 71 (23.1%) | 10 (23.8%) | | |  | |  |
| NE elevated | 159 (51.6%) | 20 (47.6%) | | |  | |  |
| DA elevated | 4 (1.3%) | 1 (2.4%) | | |  | |  |
| Multiple of the normal reference upper limit value |  |  |  |  | |  |  |
| 24-h urinary NE^*^ | 6.9 ± 18.7 | 4.8 ± 14.4 | | | 0.98 (0.94, 1.03) | | 0.424 |
| 24-h urinary E^*^ | 7.7 ± 13.6 | 6.1 ± 9.6 | | | 1.01 (0.99, 1.04) | | 0.305 |
| 24-h urinary D^*^ | 0.9 ± 0.6 | 1.0 ± 1.3 | | | 0.75 (0.38, 1.49) | | 0.413 |
| Tumor location, n (%) |  |  | | | 0.40 (0.07, 2.18) | | 0.287 |
| Unilateral | 39 (92.9%) | 283 (91.9%) | | |  | |  |
| Bilateral | 3 (7.1%) | 25 (8.1%) | | |  | |  |
| Maximum tumor diameter (cm) | 5.2 ± 2.3 | 4.7 ± 1.8 | | | 0.92 (0.72, 1.16) | | 0.472 |
| Preoperative medications, n (%) |  |  | | | 1.15 (0.75,1.76) | | 0.521 |
| *α* blockade only | 24 (57.1%) | 207 (67.2%) | | |  | |  |
| *α* blockade +CCB | 8 (19.0%) | 24 (7.8%) | | |  | |  |
| *α* blockade+*β* blockade | 5 (11.9%) | 58 (18.8%) | | |  | |  |
| *α* blockade+*β* blockade+CCB | 5 (11.9%) | 19 (6.2%) | | |  | |  |
| Duration of *α* blockade (day) | 45.5 (35, 63) | 41 (29, 59) | | | 1.00 (0.99, 1.01) | | 0.776 |
| Phenoxybenzamine (mg/day) | 30 (20, 40) | 30 (20, 30) | | | 1.00 (0.98, 1.02) | | 0.953 |
| Hemodynamic variables day before surgery |  |  | | |  | |  |
| SBP (mmHg) | 137.4 ± 17.9 | 136.3 ± 19.8 | | | 1.00 (0.96, 1.03) | | 0.787 |
| DBP (mmHg) | 85.5 ± 13.9 | 83.8 ± 13.0 | | | 1.01 (0.96, 1.06) | | 0.796 |
| HR (bpm) | 81.0 ± 14.4 | 80.2 ± 12.2 | | | 1.01 (0.97, 1.04) | | 0.612 |
| Surgical approach, n (%) |  |  | | | 2.72 (0.84, 8.75) | | 0.094 |
| laparoscopy | 36 (85.7%) | 301 (97.7%) | | |  | |  |
| Converted to open laparotomy | 6 (14.3%) | 7 (2.3%) | | |  | |  |
| Surgical duration (min) | 148.6 ± 98.4 | 113.0 ± 51.5 | | | 1.00 (0.99, 1.01) | | 0.569 |
| Blood loss(ml) | 150(50, 600) | 50 (50, 250) | | | 1.00 (1.00, 1.00) | | 0.116 |
| RBC transfusion, n (%) | 9 (21.4%) | 26 (8.4%) | | | 0.29 (0.03, 2.49) | | 0.259 |
| FFP transfusion, n (%) | 7 (16.7%) | 10 (3.2%) | | | 3.34 (0.23, 49.08) | | 0.378 |
| Hemoglobin drop (10g.L^-1^ decrease) | 2.5 ± 1.6 | 1.7 ± 1.0 | | | 1.66 (1.12, 2.47) | | 0.012 |
| Postoperative hypotension requiring vasopressors, n (%) | 32 (76.2%) | 174 (56.5%) | | | 2.03 (0.84, 4.91) | | 0.116 |

BMI, body mass index; ASA, American Society of Anesthesia; NE, norepinephrine; E, epinephrine; DA, dopamine; CCB, calcium channel blockers; SBP, systolic blood pressure; DBP, diastolic blood pressure; HR, heart rate; bpm, beat per minute; mmHg, millimeter of mercury; RBC, red blood cell; FFP, fresh frozen plasma; LOS, length of stay. Data are represented as mean ± SD, median [25th, 75th percentiles] or n (%).OR,odds ratio; CI, confidence interval.^*^multiple of the normal reference upper limit value. P value from multivariable logistic regression;

**Supplemental Table S12.**Multivariable logistic regression model for intraoperative highest systolic blood pressure over 210 mmHg.

| Factors | Myocardial injury  (n = 42) | No myocardial injury  (n = 308) | | | Adjusted OR (95% CI) | | *P* value |
| --- | --- | --- | --- | --- | --- | --- | --- |
| SBP > 210 mmHg | 19(45.2%) | 88(28.6%) | | | 2.71(1.16,6.32) | | 0.021 |
| Age, yr | 44.4 ± 14.5 | 44.6 ± 13.3 | | | 0.98(0.94, 1.02) | | 0.265 |
| Male, n (%) | 22 (52.4%) | 139 (45.1%) | | | 0.47 (0.13, 1.66) | | 0.240 |
| BMI, kg/m^2^ | 23.3 ± 3.1 | 23.9 ± 3.3 | | | 0.91 (0.80, 1.04) | | 0.172 |
| ASA physical status, n (%) |  |  | | | 1.62 (0.76, 3.43) | | 0.217 |
| 1 | 0 (0.0) | 9 (2.9%) | | |  | |  |
| 2 | 15 (35.7%) | 127 (41.2%) | | |  | |  |
| 3 | 27 (64.3%) | 172 (55.8%) | | |  | |  |
| Smoking history, n (%) | 12 (28.6%) | 51 (16.6%) | | | 1.67 (0.47,5.85） | | 0.425 |
| Alcohol use, n (%) | 10 (23.8%) | 50 (16.2%) | | | 2.10 (0.59,7.49) | | 0.254 |
| Diabetes, n (%) | 9 (21.4%) | 55 (17.9%) | | | 1.05 (0.63, 1.76) | | 0.853 |
| Hypertension, n (%) | 20 (47.6%) | 137 (44.5%) | | | 0.57 (0.30, 1.10) | | 0.092 |
| Previous ischemic heart disease or stroke, n (%) | 8 (19.0%) | 19 (6.2%) | | | 5.83 (1.67, 20.34) | | 0.006 |
| Congestive heart failure, n (%) | 4 (9.5%) | 17 (5.5%) | | | 1.87 (0.44, 8.00) | | 0.401 |
| Preoperative creatine (µmol/L) | 70.4 ± 17.4 | 67.9 ± 14.5 | | | 1.02 (0.98, 1.05) | | 0.392 |
| Preoperative 24-h urinary catecholamine elevated level |  |  | | | 0.94 (0.55,1.59) | | 0.805 |
| None | 74 (24.0%) | 11 (26.2%) | | |  | |  |
| E elevated | 71 (23.1%) | 10 (23.8%) | | |  | |  |
| NE elevated | 159 (51.6%) | 20 (47.6%) | | |  | |  |
| DA elevated | 4 (1.3%) | 1 (2.4%) | | |  | |  |
| Multiple of the normal reference upper limit value |  |  |  |  | |  |  |
| 24-h urinary NE^*^ | 6.9 ± 18.7 | 4.8 ± 14.4 | | | 0.98 (0.94, 1.03) | | 0.438 |
| 24-h urinary E^*^ | 7.7 ± 13.6 | 6.1 ± 9.6 | | | 1.01 (0.99, 1.04) | | 0.354 |
| 24-h urinary D^*^ | 0.9 ± 0.6 | 1.0 ± 1.3 | | | 0.76 (0.37, 1.53) | | 0.436 |
| Tumor location, n (%) |  |  | | | 0.41 (0.07, 2.29) | | 0.309 |
| Unilateral | 39 (92.9%) | 283 (91.9%) | | |  | |  |
| Bilateral | 3 (7.1%) | 25 (8.1%) | | |  | |  |
| Maximum tumor diameter (cm) | 5.2 ± 2.3 | 4.7 ± 1.8 | | | 0.90 (0.71, 1.15) | | 0.393 |
| Preoperative medications, n (%) |  |  | | | 1.11 (0.72,1.71) | | 0.626 |
| *α* blockade only | 24 (57.1%) | 207 (67.2%) | | |  | |  |
| *α* blockade +CCB | 8 (19.0%) | 24 (7.8%) | | |  | |  |
| *α* blockade+*β* blockade | 5 (11.9%) | 58 (18.8%) | | |  | |  |
| *α* blockade+*β* blockade+CCB | 5 (11.9%) | 19 (6.2%) | | |  | |  |
| Duration of *α* blockade (day) | 45.5 (35, 63) | 41 (29, 59) | | | 1.00 (0.99, 1.01) | | 0.761 |
| Phenoxybenzamine (mg/day) | 30 (20, 40) | 30 (20, 30) | | | 1.00 (0.98, 1.02) | | 0.975 |
| Hemodynamic variables day before surgery |  |  | | |  | |  |
| SBP (mmHg) | 137.4 ± 17.9 | 136.3 ± 19.8 | | | 1.00 (0.96, 1.03) | | 0.787 |
| DBP (mmHg) | 85.5 ± 13.9 | 83.8 ± 13.0 | | | 1.01 (0.96, 1.06) | | 0.761 |
| HR (bpm) | 81.0 ± 14.4 | 80.2 ± 12.2 | | | 1.01 (0.97, 1.04) | | 0.669 |
| Surgical approach, n (%) |  |  | | | 2.80 (0.85, 9.24) | | 0.091 |
| laparoscopy | 36 (85.7%) | 301 (97.7%) | | |  | |  |
| Converted to open laparotomy | 6 (14.3%) | 7 (2.3%) | | |  | |  |
| Surgical duration (min) | 148.6 ± 98.4 | 113.0 ± 51.5 | | | 1.00 (0.99, 1.01) | | 0.537 |
| Blood loss(ml) | 150(50, 600) | 50 (50, 250) | | | 1.00 (1.00, 1.00) | | 0.073 |
| RBC transfusion, n (%) | 9 (21.4%) | 26 (8.4%) | | | 0.22 (0.02, 1.98) | | 0.176 |
| FFP transfusion, n (%) | 7 (16.7%) | 10 (3.2%) | | | 3.07 (0.20, 47.77) | | 0.423 |
| Hemoglobin drop (10g.L^-1^ decrease) | 2.5 ± 1.6 | 1.7 ± 1.0 | | | 1.69 (1.13, 2.53) | | 0.011 |
| Postoperative hypotension requiring vasopressors, n (%) | 32 (76.2%) | 174 (56.5%) | | | 1.99 (0.82, 4.81) | | 0.127 |

BMI, body mass index; ASA, American Society of Anesthesia; NE, norepinephrine; E, epinephrine; DA, dopamine; CCB, calcium channel blockers; SBP, systolic blood pressure; DBP, diastolic blood pressure; HR, heart rate; bpm, beat per minute; mmHg, millimeter of mercury; RBC, red blood cell; FFP, fresh frozen plasma; LOS, length of stay. Data are represented as mean ± SD, median [25th, 75th percentiles] or n (%). OR,odds ratio; CI, confidence interval.^*^multiple of the normal reference upper limit value. P value from multivariable logistic regression;

**Supplemental Table S13.**Multivariable logistic regression model for intraoperative highest systolic blood pressure over 220 mmHg.

| Factors | Myocardial injury  (n = 42) | No myocardial injury  (n = 308) | | | Adjusted OR (95% CI) | | *P* value |
| --- | --- | --- | --- | --- | --- | --- | --- |
| SBP > 220 mmHg | 24(57.1%) | 125(40.6%) | | | 3.03(1.18,7.75) | | 0.021 |
| Age, yr | 44.4 ± 14.5 | 44.6 ± 13.3 | | | 0.98(0.94, 1.02) | | 0.249 |
| Male, n (%) | 22 (52.4%) | 139 (45.1%) | | | 0.50 (0.14, 1.75) | | 0.275 |
| BMI, kg/m^2^ | 23.3 ± 3.1 | 23.9 ± 3.3 | | | 0.92 (0.80, 1.05) | | 0.221 |
| ASA physical status, n (%) |  |  | | | 1.74 (0.82, 3.73) | | 0.151 |
| 1 | 0 (0.0) | 9 (2.9%) | | |  | |  |
| 2 | 15 (35.7%) | 127 (41.2%) | | |  | |  |
| 3 | 27 (64.3%) | 172 (55.8%) | | |  | |  |
| Smoking history, n (%) | 12 (28.6%) | 51 (16.6%) | | | 1.47 (0.43,5.06） | | 0.542 |
| Alcohol use, n (%) | 10 (23.8%) | 50 (16.2%) | | | 2.50 (0.69,9.07) | | 0.164 |
| Diabetes, n (%) | 9 (21.4%) | 55 (17.9%) | | | 1.08 (0.65, 1.82) | | 0.759 |
| Hypertension, n (%) | 20 (47.6%) | 137 (44.5%) | | | 0.56 (0.29, 1.08) | | 0.084 |
| Previous ischemic heart disease or stroke, n (%) | 8 (19.0%) | 19 (6.2%) | | | 5.16 (1.49, 17.89) | | 0.010 |
| Congestive heart failure, n (%) | 4 (9.5%) | 17 (5.5%) | | | 1.82 (0.43, 7.68) | | 0.413 |
| Preoperative creatine (µmol/L) | 70.4 ± 17.4 | 67.9 ± 14.5 | | | 1.01 (0.98, 1.05) | | 0.460 |
| Preoperative 24-h urinary catecholamine elevated level |  |  | | | 0.95 (0.56,1.62) | | 0.862 |
| None | 74 (24.0%) | 11 (26.2%) | | |  | |  |
| E elevated | 71 (23.1%) | 10 (23.8%) | | |  | |  |
| NE elevated | 159 (51.6%) | 20 (47.6%) | | |  | |  |
| DA elevated | 4 (1.3%) | 1 (2.4%) | | |  | |  |
| Multiple of the normal reference upper limit value |  |  |  |  | |  |  |
| 24-h urinary NE^*^ | 6.9 ± 18.7 | 4.8 ± 14.4 | | | 0.98 (0.94, 1.03) | | 0.475 |
| 24-h urinary E^*^ | 7.7 ± 13.6 | 6.1 ± 9.6 | | | 1.01 (0.99, 1.04) | | 0.324 |
| 24-h urinary D^*^ | 0.9 ± 0.6 | 1.0 ± 1.3 | | | 0.71 (0.33, 1.51) | | 0.372 |
| Tumor location, n (%) |  |  | | | 0.41 (0.07, 2.28) | | 0.307 |
| Unilateral | 39 (92.9%) | 283 (91.9%) | | |  | |  |
| Bilateral | 3 (7.1%) | 25 (8.1%) | | |  | |  |
| Maximum tumor diameter (cm) | 5.2 ± 2.3 | 4.7 ± 1.8 | | | 0.92 (0.72, 1.17) | | 0.487 |
| Preoperative medications, n (%) |  |  | | | 1.12 (0.72,1.74) | | 0.604 |
| *α* blockade only | 24 (57.1%) | 207 (67.2%) | | |  | |  |
| *α* blockade +CCB | 8 (19.0%) | 24 (7.8%) | | |  | |  |
| *α* blockade+*β* blockade | 5 (11.9%) | 58 (18.8%) | | |  | |  |
| *α* blockade+*β* blockade+CCB | 5 (11.9%) | 19 (6.2%) | | |  | |  |
| Duration of *α* blockade (day) | 45.5 (35, 63) | 41 (29, 59) | | | 1.00 (0.99, 1.01) | | 0.868 |
| Phenoxybenzamine (mg/day) | 30 (20, 40) | 30 (20, 30) | | | 1.00 (0.98, 1.02) | | 0.857 |
| Hemodynamic variables day before surgery |  |  | | |  | |  |
| SBP (mmHg) | 137.4 ± 17.9 | 136.3 ± 19.8 | | | 1.00 (0.96, 1.03) | | 0.784 |
| DBP (mmHg) | 85.5 ± 13.9 | 83.8 ± 13.0 | | | 1.00 (0.96, 1.05) | | 0.853 |
| HR (bpm) | 81.0 ± 14.4 | 80.2 ± 12.2 | | | 1.01 (0.97, 1.04) | | 0.659 |
| Surgical approach, n (%) |  |  | | | 2.51 (0.78, 8.04) | | 0.123 |
| laparoscopy | 36 (85.7%) | 301 (97.7%) | | |  | |  |
| Converted to open laparotomy | 6 (14.3%) | 7 (2.3%) | | |  | |  |
| Surgical duration (min) | 148.6 ± 98.4 | 113.0 ± 51.5 | | | 1.00 (0.99, 1.01) | | 0.586 |
| Blood loss(ml) | 150(50, 600) | 50 (50, 250) | | | 1.00 (1.00, 1.00) | | 0.133 |
| RBC transfusion, n (%) | 9 (21.4%) | 26 (8.4%) | | | 0.32 (0.04, 2.69) | | 0.297 |
| FFP transfusion, n (%) | 7 (16.7%) | 10 (3.2%) | | | 3.37 (0.23, 49.09) | | 0.374 |
| Hemoglobin drop (10g.L^-1^ decrease) | 2.5 ± 1.6 | 1.7 ± 1.0 | | | 1.74 (1.16, 2.62) | | 0.008 |
| Postoperative hypotension requiring vasopressors, n (%) | 32 (76.2%) | 174 (56.5%) | | | 2.00 (0.82, 4.86) | | 0.126 |

BMI, body mass index; ASA, American Society of Anesthesia; NE, norepinephrine; E, epinephrine; DA, dopamine; CCB, calcium channel blockers; SBP, systolic blood pressure; DBP, diastolic blood pressure; HR, heart rate; bpm, beat per minute; mmHg, millimeter of mercury; RBC, red blood cell; FFP, fresh frozen plasma; LOS, length of stay. Data are represented as mean ± SD, median [25th, 75th percentiles] or n (%).OR,odds ratio; CI, confidence interval.^*^multiple of the normal reference upper limit value. P value from multivariable logistic regression;

**Supplemental Table S14.** Summary of multivariable logistic regression models for the duration of intraoperative heart rate.

| Factors | Myocardial injury  (n = 42) | No Myocardial injury  (n = 308) | Unadjusted OR (95% CI) | *P* | Adjusted OR (95% CI) | *P* ^*^ |
| --- | --- | --- | --- | --- | --- | --- |
| Duration of HR > 100 bpm |  |  |  |  |  |  |
| > 1min | 35 (83.3%) | 198 (64.3%) | 2.78 (1.19, 6.46) | 0.018 | 1.93 (0.72, 5.14) | 0.189 |
| > 5min | 27 (64.3%) | 147 (47.7%) | 1.97 (1.01, 3.85) | 0.047 | 1.52 (0.64, 3.60) | 0.339 |
| > 10min | 22 (52.4%) | 104 (33.8%) | 2.16 (1.13, 4.13) | 0.020 | 1.58 (0.69, 3.61) | 0.278 |
| > 15min | 20 (47.6%) | 84 (27.3%) | 2.42 (1.25, 4.67) | 0.008 | 1.76 (0.75, 4.14) | 0.193 |
| > 20min | 18 (42.9%) | 66 (21.4%) | 2.75 (1.40, 5.37) | 0.003 | 1.89 (0.77, 4.63) | 0.166 |
| Duration of HR > 105 bpm |  |  |  |  |  |  |
| > 1min | 32 (76.2%) | 162 (52.6%) | 2.88(1.37, 6.07) | 0.005 | 2.04(0.85,4.89) | 0.111 |
| > 5min | 22 (52.4%) | 97 (31.5%) | 2.39(1.24, 4.59) | 0.009 | 1.85(0.79,4.33) | 0.153 |
| > 10min | 19 (45.2%) | 66 (21.4%) | 3.03(1.56, 5.89) | 0.001 | 2.09(0.83,5.29) | 0.118 |
| > 15min | 17 (40.5%) | 45 (14.6%) | 3.97 (1.98,7.94） | <0.001 | 3.69(1.32,10.35) | 0.013 |
| > 20min | 17 (40.5%) | 31 (10.1%) | 6.08 (2.96,12.47) | <0.001 | 5.72(2.00,16.34) | 0.001 |
| Duration of HR > 110bpm |  |  |  |  |  |  |
| > 1min | 13 (31.0%) | 26 (8.4%) | 3.05(1.53,6.10) | 0.002 | 2.66(1.12,6.27) | 0.026 |
| > 5min | 20 (47.6%) | 63 (20.5%) | 3.54(1.82,6.88) | <0.001 | 3.56(1.41,8.94) | 0.007 |
| > 10min | 16 (38.1%) | 36 (11.7%) | 4.65(2.28,9.49) | <0.001 | 5.76(1.95,17.01) | 0.002 |
| > 15min | 13 (31.0%) | 26 (8.4%) | 4.86(2.26,10.48) | <0.001 | 3.85(1.29,11.50) | 0.016 |
| > 20min | 11 (26.2%) | 20 (6.5%) | 5.11(2.24,11.64) | <0.001 | 3.65(1.09,12.25) | 0.036 |
| Duration of HR > 115bpm |  |  |  |  |  |  |
| > 1min | 24 (57.1%) | 89 (28.9%) | 3.28(1.69,6.34) | <0.001 | 3.55(1.49,8.49) | 0.004 |
| > 5min | 13 (31.0%) | 39 (12.7%) | 3.09(1.48,6.45) | 0.003 | 3.14(1.08,9.17) | 0.036 |
| > 10min | 10 (23.8%) | 21 (6.8%) | 4.27(1.85,9.86) | 0.001 | 4.18(1.15,15.14) | 0.030 |
| > 15min | 8 (19.0%) | 16 (5.2%) | 4.29(1.71,10.78) | 0.002 | 4.36(1.00,19.05) | 0.050 |
| > 20min | 7 (16.7%) | 8 (2.6%) | 7.5(2.56,21.93) | <0.001 | 7.68(1.11,53.13) | 0.039 |
| Duration of HR > 120bpm |  |  |  |  |  |  |
| > 1min | 19 (45.2%) | 61 (19.8%) | 3.34(1.71,6.53) | <0.001 | 3.68(1.51,8.96) | 0.004 |
| > 5min | 10 (23.8%) | 23 (7.5%) | 3.87(1.69,8.86) | 0.001 | 3.75(1.06,13.22) | 0.040 |
| > 10min | 6 (14.3%) | 10 (3.2%) | 4.97(1.70,14.47) | 0.003 | 3.38(0.59,19.26) | 0.170 |
| > 15min | 5 (11.9%) | 4 (1.3%) | 10.27（2.64,39.95） | 0.001 | 12.10(1.13,129.07) | 0.039 |
| > 20min | 5 (11.9%) | 4 (1.3%) | 10.27（2.64,39.95） | 0.001 | 12.10(1.13,129.07) | 0.039 |

HR, heart rate; bpm, beat per minute; OR,odds ratio; CI, confidence interval. Data are represented as n (%). *P* from univariable logistic regression; ^*^ *P* from multivariable logistic regression;

**Supplemental Table S15.**Multivariable logistic regression model for intraoperative highest SBP > 210 mmHg and HR >115 bpm last for over 1 minute

| Factors | Myocardial injury  (n = 42) | No myocardial injury  (n = 308) | | | Adjusted OR (95% CI) | | *P* value |
| --- | --- | --- | --- | --- | --- | --- | --- |
| HR >115 bpm over 1mins | 24(57.1%) | 89(28.9%) | | | 2.66(1.08,6.60) | | 0.034 |
| SBP > 210 mmHg over 1mins | 16(38.1%) | 48(15.6%) | | | 3.78(1.47,9.73) | | 0.006 |
| Age, yr | 44.4 ± 14.5 | 44.6 ± 13.3 | | | 0.98(0.94, 1.03) | | 0.446 |
| Male, n (%) | 22 (52.4%) | 139 (45.1%) | | | 0.50 (0.14, 1.78) | | 0.283 |
| BMI, kg/m^2^ | 23.3 ± 3.1 | 23.9 ± 3.3 | | | 0.92 (0.80, 1.06) | | 0.241 |
| ASA physical status, n (%) |  |  | | | 1.63 (0.74, 3.61) | | 0.228 |
| 1 | 0 (0.0) | 9 (2.9%) | | |  | |  |
| 2 | 15 (35.7%) | 127 (41.2%) | | |  | |  |
| 3 | 27 (64.3%) | 172 (55.8%) | | |  | |  |
| Smoking history, n (%) | 12 (28.6%) | 51 (16.6%) | | | 1.93 (0.53,7.05） | | 0.322 |
| Alcohol use, n (%) | 10 (23.8%) | 50 (16.2%) | | | 1.87 (0.49,7.12) | | 0.361 |
| Diabetes, n (%) | 9 (21.4%) | 55 (17.9%) | | | 1.01 (0.60, 1.72) | | 0.959 |
| Hypertension, n (%) | 20 (47.6%) | 137 (44.5%) | | | 0.51 (0.25, 1.04) | | 0.066 |
| Previous ischemic heart disease or stroke, n (%) | 8 (19.0%) | 19 (6.2%) | | | 3.98 (1.11, 14.32) | | 0.034 |
| Congestive heart failure, n (%) | 4 (9.5%) | 17 (5.5%) | | | 1.80 (0.41, 7.96) | | 0.438 |
| Preoperative creatine (µmol/L) | 70.4 ± 17.4 | 67.9 ± 14.5 | | | 1.02 (0.98, 1.05) | | 0.400 |
| Preoperative 24-h urinary catecholamine elevated level |  |  | | | 0.98 (0.57,1.69) | | 0.946 |
| None | 74 (24.0%) | 11 (26.2%) | | |  | |  |
| E elevated | 71 (23.1%) | 10 (23.8%) | | |  | |  |
| NE elevated | 159 (51.6%) | 20 (47.6%) | | |  | |  |
| DA elevated | 4 (1.3%) | 1 (2.4%) | | |  | |  |
| Multiple of the normal reference upper limit value |  |  |  |  | |  |  |
| 24-h urinary NE^*^ | 6.9 ± 18.7 | 4.8 ± 14.4 | | | 0.98 (0.94, 1.03) | | 0.384 |
| 24-h urinary E^*^ | 7.7 ± 13.6 | 6.1 ± 9.6 | | | 1.01 (0.98, 1.04) | | 0.393 |
| 24-h urinary D^*^ | 0.9 ± 0.6 | 1.0 ± 1.3 | | | 0.69 (0.32, 1.51) | | 0.357 |
| Tumor location, n (%) |  |  | | | 0.34 (0.06, 2.04) | | 0.235 |
| Unilateral | 39 (92.9%) | 283 (91.9%) | | |  | |  |
| Bilateral | 3 (7.1%) | 25 (8.1%) | | |  | |  |
| Maximum tumor diameter (cm) | 5.2 ± 2.3 | 4.7 ± 1.8 | | | 0.89 (0.70, 1.13) | | 0.336 |
| Preoperative medications, n (%) |  |  | | | 1.21 (0.77,1.89) | | 0.409 |
| *α* blockade only | 24 (57.1%) | 207 (67.2%) | | |  | |  |
| *α* blockade +CCB | 8 (19.0%) | 24 (7.8%) | | |  | |  |
| *α* blockade+*β* blockade | 5 (11.9%) | 58 (18.8%) | | |  | |  |
| *α* blockade+*β* blockade+CCB | 5 (11.9%) | 19 (6.2%) | | |  | |  |
| Duration of *α* blockade (day) | 45.5 (35, 63) | 41 (29, 59) | | | 1.00 (0.99, 1.01) | | 0.873 |
| Phenoxybenzamine (mg/day) | 30 (20, 40) | 30 (20, 30) | | | 1.00 (0.98, 1.03) | | 0.540 |
| Hemodynamic variables day before surgery |  |  | | |  | |  |
| SBP (mmHg) | 137.4 ± 17.9 | 136.3 ± 19.8 | | | 0.99 (0.95, 1.03) | | 0.620 |
| DBP (mmHg) | 85.5 ± 13.9 | 83.8 ± 13.0 | | | 1.01 (0.96, 1.06) | | 0.722 |
| HR (bpm) | 81.0 ± 14.4 | 80.2 ± 12.2 | | | 1.00 (0.97, 1.04) | | 0.997 |
| Surgical approach, n (%) |  |  | | | 3.21 (0.97, 10.66) | | 0.056 |
| laparoscopy | 36 (85.7%) | 301 (97.7%) | | |  | |  |
| Converted to open laparotomy | 6 (14.3%) | 7 (2.3%) | | |  | |  |
| Surgical duration (min) | 148.6 ± 98.4 | 113.0 ± 51.5 | | | 1.00 (0.98, 1.01) | | 0.345 |
| Blood loss(ml) | 150(50, 600) | 50 (50, 250) | | | 1.00 (1.00, 1.00) | | 0.103 |
| RBC transfusion, n (%) | 9 (21.4%) | 26 (8.4%) | | | 0.23 (0.02, 2.18) | | 0.198 |
| FFP transfusion, n (%) | 7 (16.7%) | 10 (3.2%) | | | 2.85 (0.16, 51.89) | | 0.480 |
| Hemoglobin drop (10g.L^-1^ decrease) | 2.5 ± 1.6 | 1.7 ± 1.0 | | | 1.79 (1.17, 2.73) | | 0.007 |
| Postoperative hypotension requiring vasopressors, n (%) | 32 (76.2%) | 174 (56.5%) | | | 2.35 (0.92, 6.01) | | 0.075 |

BMI, body mass index; ASA, American Society of Anesthesia; NE, norepinephrine; E, epinephrine; DA, dopamine; CCB, calcium channel blockers; SBP, systolic blood pressure; DBP, diastolic blood pressure; HR, heart rate; bpm, beat per minute; mmHg, millimeter of mercury; RBC, red blood cell; FFP, fresh frozen plasma; LOS, length of stay. Data are represented as mean ± SD, median [25th, 75th percentiles] or n (%).OR,odds ratio; CI, confidence interval.^*^multiple of the normal reference upper limit value. P value from multivariable logistic regression;

**Supplemental Table S16.** Summary multivariable logistic regression models for the duration of intraoperative highest systolic blood pressure lasted for 1 minute.

| Factors | Myocardial injury  (n = 42) | No Myocardial injury  (n = 308) | Unadjusted OR (95% CI) | *P* | Adjusted OR (95% CI) | *P* ^*^ |
| --- | --- | --- | --- | --- | --- | --- |
| Duration of SBP > 160 mmHg |  |  |  |  |  |  |
| > 1min | 36 (85.7%) | 256 (83.1%) | 1.22 (0.49, 3.04) | 0.671 | 0.71 (0.20, 2.51) | 0.600 |
| Duration of SBP > 180 mmHg |  |  |  |  |  |  |
| > 1min | 27 (64.3%) | 154 (50.0%) | 1.8(0.92, 3.52) | 0.085 | 1.53(0.61,3.83) | 0.363 |
| Duration of SBP > 200 mmHg |  |  |  |  |  |  |
| > 1min | 20 (47.6%) | 77 (25.0%) | 2.73 (1.41,5.27) | 0.003 | 3.44(1.43,8.32) | 0.006 |
| Duration of SBP > 210 mmHg |  |  |  |  |  |  |
| > 1min | 16 (38.1%) | 48 (15.6%) | 3.33(1.66, 6.68) | 0.001 | 3.78(1.47,9.73) | 0.006 |
| Duration of SBP > 220 mmHg |  |  |  |  |  |  |
| > 1min | 10 (23.8%) | 28 (9.1%) | 3.13(1.39,7.02) | 0.006 | 3.92(1.29, 11.95) | 0.016 |

SBP, systolic blood pressure; mmHg, millimeter of mercury; OR,odds ratio; CI, confidence interval. Data are represented as n (%). *P* from univariable logistic regression; *P* from multivariable logistic regression, ^*^ *P* from multivariable logistic regression.
